# Construction of ovarian metastasis‐related immune signature predicting prognosis of gastric cancer patients

**DOI:** 10.1002/cam4.4857

**Published:** 2022-05-27

**Authors:** Jianpeng Gao, Shiying Huo, Yu Zhang, Zhenxiong Zhao, Hongda Pan, Xiaowen Liu

**Affiliations:** ^1^ Department of Gastric Surgery Fudan University Shanghai Cancer Center Shanghai China; ^2^ Department of Oncology Shanghai Medical College, Fudan University Shanghai China

**Keywords:** gastric cancer, immune‐related gene, ovarian metastasis, prognosis

## Abstract

**Background:**

Ovarian metastasis (OM) results in poor survival of gastric cancer (GC) patients. While immunotherapy has emerged as a promising approach for late‐stage GC, validated immune‐related prognostic signatures still remain in need. In this study, we constructed an ovarian metastasis‐ and immune‐related prognostic signature (OMIRPS), characterized the molecular and immune features of OMIRPS‐categorized subgroups and predicted their potential response to immunotherapy.

**Methods:**

Three individual cohorts were used to construct and evaluate OMIRPS: RNA‐seq of matched primary GC and OM from Fudan University Shanghai Cancer Center (FUSCC) (discovery cohort, *n* = 4), The Cancer Genome Atlas (TCGA) (training cohort, *n* = 544) and GSE84437 (validation cohort, *n* = 433). Differentially expressed genes (DEGs) identified between primary GC and OM and immune‐related genes (IRGs) from the ImmPort and InnateDB databases were used to identify immune‐related prognostic hub genes, which were further used to construct OMIRPS by using LASSO regression analysis. Prognosis, molecular characteristics, immune features, and differential immunotherapy efficacy between different OMIRPS subgroups were analyzed.

**Results:**

Functional analyses of DEGs revealed the significance of immune‐related signatures and pathways in the OM. Immune‐related prognostic hub genes including *TNFRSF18*, *CARD11*, *BCL11B*, *NRP1*, *BNIP3L,* and *ATF3* were utilized to construct OMIRPS, which was identified as an independent prognostic factor. Comprehensive analyses unveiled the distinctive molecular and immune characteristics of OMIRPS‐high and ‐low subgroup in regard to enriched pathways, mutation rate, tumor mutation burden, microsatellite instability status, infiltrated immune cell, immune exclusion score, and the prediction of immunotherapy efficacy. Additionally, OMIRPS was associated with Immune Subtypes with borderline significance.

**Conclusions:**

RNA‐seq of paired primary and ovarian metastatic tumors unveiled the significance of immune‐related pathways and tumor immune microenvironment in OM. OMIRPS served as a promising biomarker to predict the prognosis of GC patients and distinguish the molecular features, immune characteristics, and efficacy of immunotherapy between different subgroups.

## Graphical abstract

Schematic layout of constructing ovarian metastasis‐ and immune‐related prognostic signature (OMIRPS). Construction and validation of OMIRPS was based on comprehensive bioinformatic analyses of RNA‐seq data from our own cohort in combined with public datasets.

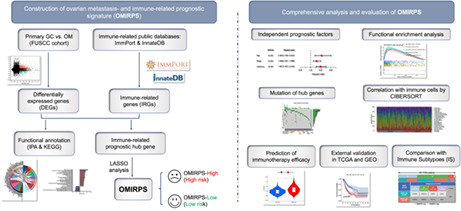

## 1 INTRODUCTION

Gastric cancer (GC) is the third leading cause of cancer‐related deaths worldwide and distant metastasis is one of the largest threats to its poor prognosis.^1^ Ovarian metastasis (OM) is one unique type of metastasis in female patients, with its incidence ranging from 0.3% to 6.7%.^2^, ^3^ Despite the combined efforts of mastectomy and systemic chemotherapy, the median survival period remains <19 months,^4^, ^5^ indicating the limited efficacy of current therapeutics and the urgent need to develop new approaches to the treatment of OM.

In recent years, immunotherapy has emerged as a promising method in cancer treatment.^6^ Notably, monoclonal antibodies targeting immune checkpoints such as programmed death‐1 (PD‐1) and programmed death‐ligand 1 (PD‐L1) have shown efficacy in melanoma, non‐small cell lung cancer, renal cell carcinoma, as well as gastrointestinal malignancy.^7^ With respect to GC, clinical trials have demonstrated antitumor activity with PD‐1/PD‐L1 inhibitors,^8^ with the milestone clinical trial Checkmate 649 leading to the Food and Drug Administration approval of the PD‐1 inhibitor nivolumab for advanced or metastatic GC.^9^ However, immune checkpoint inhibitor (ICI)‐based immunotherapy has not been widely applied in the treatment of GC patients with OM to date. Moreover, the response rate of GC patients to ICI is reportedly low,^10^ which could be partially explained by the distinctive molecular and immune characteristics such as tumor immune microenvironment (TIME) of different patients.^11^ To identify the subgroup of GC patients who are more likely to benefit from immunotherapy, immune‐related predictive models such as TMEscore have been developed.^12^, ^13^ Nevertheless, effective immune‐related prognostic and therapeutic indicators have not been constructed in GC patients with OM so far.

In the present study, we aimed to establish an OM‐related signature which could predict the prognosis of GC patients and their response to immunotherapy. RNA‐seq analyses of DEGs between paired primary GC and OM from FUSCC cohort uncovered the significance of immune‐related signatures and pathways. Based on DEGs and public databases‐derived immune‐related genes (IRGs), we identified a total number of 19 immune‐related hub prognostic genes. We further utilized a LASSO regression to construct an OM‐ and immune‐related prognostic signature (OMIRPS). We validated the prognostic value of OMIRPS in both TCGA‐STAD and GSE84437 cohort, characterized the molecular and immunological features of different OMRIPS subgroups and evaluated its predictive ability of immunotherapy efficacy. We compared the OMIRPS scoring with other classification systems and unveiled that OMIRPS was independent from TNM staging and was associated with Immune Subtypes (IS) with borderline significance. These results suggested that OMIRPS, which was originated from the bioinformatic studies of FUSCC cohort and public databases (TCGA‐STAD and GSE84437 cohort), served as a promising OM‐related immune biomarker for the prediction of patient prognosis and immunotherapy efficacy.

## 2 MATERIALS AND METHODS

### 2.1 RNA sequencing of paired primary GCs and OMs from Fudan University Shanghai Cancer Center

The formalin‐fixed paraffin‐embedded (FFPE) tissues of primary gastric tumors and matching metastatic ovarian lesions (*n* = 4) were collected from patients undergoing extended radical gastrectomy without neoadjuvant chemotherapy or radiotherapy between 2016 and 2020 at the Fudan University Shanghai Cancer Center (FUSCC in short). Their tissues were used for RNA sequencing (RNA‐seq in short), and this dataset was used as the discovery cohort. Histological and tumor cellularity were determined (tumor cellularity over 60%) by two individual pathologists before sequencing. The 8th edition of UICC TNM classification was applied to determine the pathological stage of patients. This study was approved by the Ethics Committee of FUSCC, and informed consents were received from all patients. For RNA‐seq, RNAstormTM FFPE kit (CELLDATA) was used to isolate total RNA. SMARTer Stranded Total RNA‐Seq Kit‐Pico Input Mammalian Library preparation kit (Clontech) was used to prepare a strand‐specific RNA‐seq library. Qubit fluorometer (Thermo Fisher Scientific) and Qsep100 (BiOptic) were used to check the quality of library. Illumina sequencing platform with 150 bp paired‐end run metrics was used for performing RNA‐seq.

### 2.2 Data analysis of RNA‐seq

FastQC was applied to filter raw reads and remove low quality bases and adaptor sequences. HISAT2 was applied to map the reads to the GRCh38 human genome assembly. FPKM (Fragments Per Kilobase of transcript per Million mapped reads) was applied for the calculation of gene expression level by normalizing gene counts from feature counts. The Pearson correlation coefficient was calculated to compare the global gene expression in the samples. The analysis was carried out by R software (version 3.6.1) with the “limma” package, and the significance threshold was set as |log2[fold change (FC)]| > 1, and False Discovery Rates (FDR) <0.05.

### 2.3 Data acquisition and preprocessing

RNA‐seq and somatic mutation data of 407 GC samples, including 375 cancer samples and 32 para‐cancer samples, and their corresponding clinicopathological information were downloaded from the Stomach Adenocarcinoma dataset of The Cancer Genome Atlas (TCGA) (https://portal.gdc.cancer.gov/projects/TCGA‐STAD). In addition, microarray expression profiling data of 433 GC samples (GSE84437) and the clinicopathological information were downloaded from the GEO database (https://www.ncbi.nlm.nih.gov/geo/). The TCGA dataset served as the training cohort and the GSE84437 dataset was used as a validation cohort to verify the predictive ability of the ovarian metastasis‐related immune and prognostic signature (OMIRPS). The TCGA molecular classification and Immune Subtypes (IS) of GC samples were extracted from the supplementary files of the article of Thorsson et al.^14^ “ComBat” tool from the “sva” package was applied to adjust for systematic batch effects between TCGA and GEO dataset.

### 2.4 Immune‐related genes (IRGs) lists

Two lists of IRGs were downloaded from the ImmPort (https://www.immport.org/shared/home)^15^ and InnateDB (https://www.innateDBdb.com/) databases.^16^ A final list of IRGs was created by combining ImmPort and InnateDB lists and removing the overlapped genes. Differential expressed IRGs (DEIRGs) were selected by intersecting the list of DEGs and the list of IRG.

### 2.5 Functional annotation

To explore the biological functions of DEIRGs, Gene Ontology (GO) with biological process (BP) analysis and Kyoto Encyclopedia of Genes and Genomes (KEGG) pathway analyses were performed by using the “clusterProfiler” package in R software. To determine the signaling pathway of the gene set of different groups, a gene set enrichment analysis (GSEA) based on the Hallmark gene sets was carried out with the “GSVA” package of R. DEGs between paired primary and ovarian metastatic lesions were submitted for canonical pathway analysis by QIAGEN Ingenuity Pathway Analysis (IPA). Gene lists containing the gene probe set IDs and corresponding *p* values were uploaded to the IPA software (Ingenuity Systems; www.ingenuity.com/) to identify significantly activated and inhibited signaling pathways between samples. The −log (*p* value) > 2 and |*Z* score| > 2 were used as the threshold of significance, with a Z‐score > 2 indicating significant activation while a *Z*‐score < −2 indicating significant inhibition.

### 2.6 Construction and validation of ovarian metastasis‐ and immune‐related and prognostic signature (OMIRPS)

A univariate Cox proportional hazards regression analysis was conducted to identify prognostic hub genes associated with overall survival (OS). A least absolute shrinkage and selection operator (LASSO) Cox regression model were performed on prognostic hub genes with “glmnet” and “survival” packages. The coefficient for each gene was calculated, and genes that remained in the model were used to construct the ovarian metastasis‐ and immune‐related and prognostic signature (OMIRPS). The OMIRPS score was calculated by summing up the products of gene expressions and their corresponding coefficients. Patients were divided into OMIRPS‐high or OMIRPS‐low groups according to its median score. A Kaplan–Meier survival analysis with log‐rank test was performed with the R package “survminer” to reveal the survival difference between different OMIRPS subgroups in both training and validation cohorts. A time‐dependent receiver operating characteristic (ROC) curve analysis was conducted using the “survivalROC” package. The values of the area under the curve (AUC) at 1, 3, and 5 years were calculated to determine the prognostic predicting ability of OMIRPS. A multivariate Cox proportional hazards regression analysis was conducted to identify the independent prognostic predictor using the “survival” package. A risk heatmap comparing OMIRPS score, OS status and time, and gene expression were created using the R package “pheatmap.”

### 2.7 Somatic variants analysis

Somatic mutation data were downloaded from the Stomach Adenocarcinoma dataset of The Cancer Genome Atlas (TCGA) (https://portal.gdc.cancer.gov/projects/TCGA‐STAD). Somatic mutations of prognostic hub genes were analyzed by using the “maftools” package of R. Top 20 mutated genes were compared between IRPS‐risk and IRPS‐low groups.

### 2.8 Immune cell infiltration analysis

CIBERSORT (https://cibersort.stanford.edu/) is a computational method for quantifying cell fractions from bulk tissue gene expression profiles. We used the CIBERSORT to estimate the proportion of 22 types of immune cells in GC sample in TCGA cohort. The difference of immune cells abundance between OMIRPS‐high and OMIRPS‐low groups was analyzed. The prognostic significance of immune cells was evaluated through a Kalan–Meier analysis.

### 2.9 Prediction of immunotherapy efficacy

Tumor Immune Dysfunction and Exclusion (TIDE, http://tide.dfci.harvard.edu/) is a computational framework developed to evaluate the potential of tumor immune escape from the gene expression profiles of cancer samples. The TIDE score computed for each tumor samples can serve as a surrogate biomarker to predict the response to immune checkpoint blockade, including anti‐PD1 and anti‐CTLA4 for multiple types of cancer. The TIDE score along with immune dysfunction and exclusion scores were compared between OMIRPS‐high and OMIRPS‐low groups. The correlation between OMIRPS score and expression levels of immune checkpoint molecules (PD‐1, PD‐L1, CTLA4, and LAG3) were analyzed and visualized using the “limma”, “reshape2”, and “ggpubr” packages.

### 2.10 Statistical analysis

Statistical analyses were conducted using R (version 4.1.0). Continuous variables were presented as mean standard error of the mean (SEM) and were compared with Student's *t*‐tests or Wilcoxon rank‐sum tests. Categorical data were compared using the chi‐square test. A *p* value <0.05 was considered to be significant. Statistical significance is shown as *p < 0.05, **p < 0.01, ***p < 0.001.

## 3 RESULTS

### 3.1 Identification and functional annotation of differentially expressed immune‐related genes (DEIRGs) between primary gastric tumors and matching ovarian metastases

**FIGURE 1:**
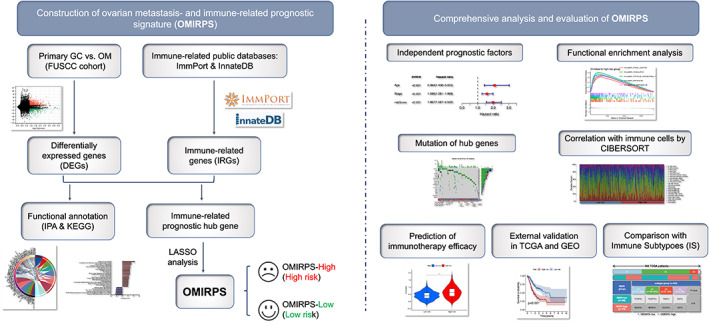
Schematic layout of constructing ovarian metastasis‐ and immune‐related prognostic signature (OMIRPS).

**FIGURE 2:**
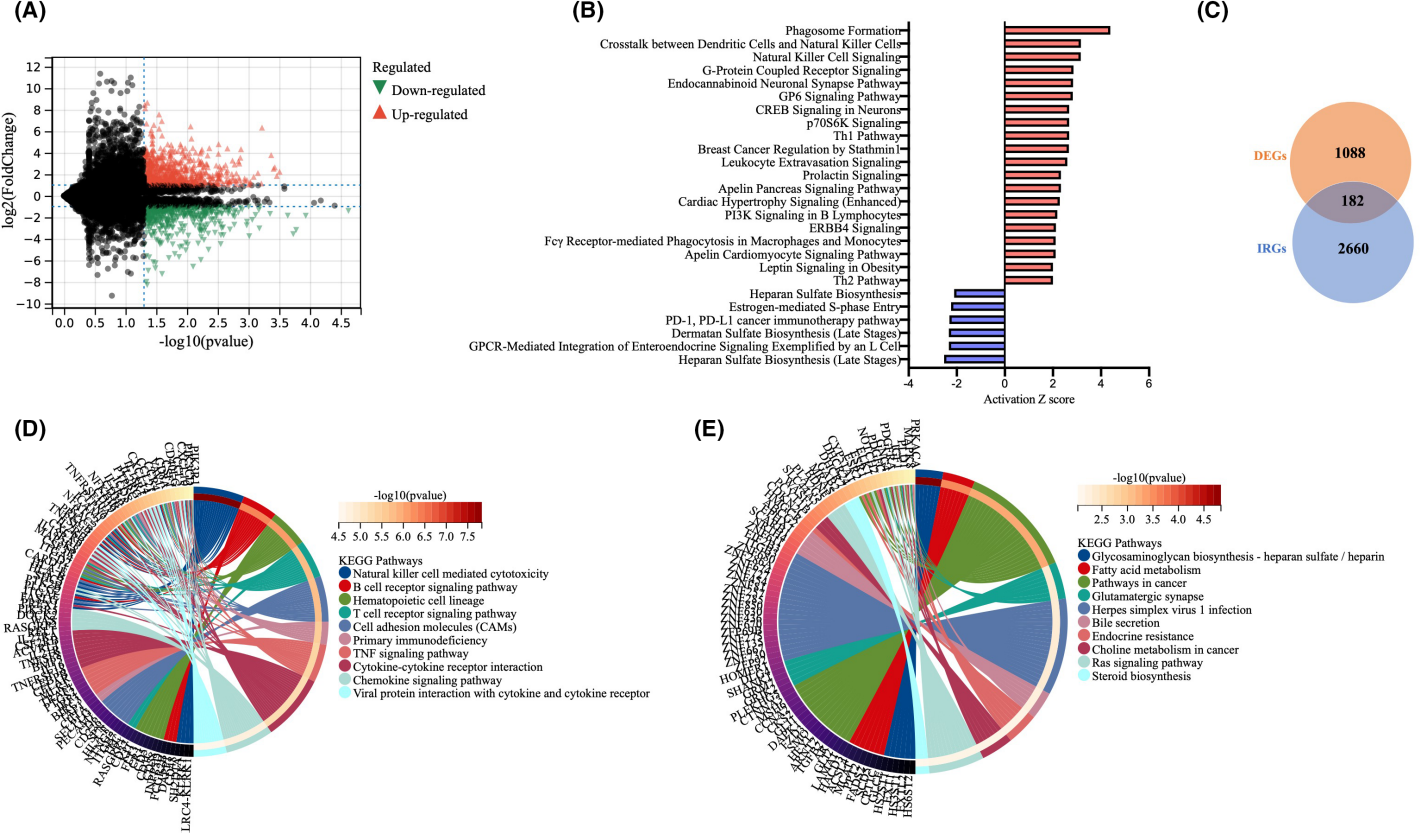
Analysis of differentially expressed genes between paired primary and ovarian metastatic lesions of four GC patients from FUSCC cohort. (A) Volcano plot of log2 fold change and ‐log10 (FDR) of differentially expressed genes between primary GC and OM. Red dots represent upregulated gene and green dots represent up‐ and downregulated genes in primary GC in comparison with OM. (B) The most changed signaling pathways between primary GC and OM revealed by the canonical pathway analysis of Ingenuity Pathway Analysis (IPA), which were denoted by the horizontal bars based on the *Z*‐scores. *Z* score > 2 (red bars) and <−2 (blue bars) indicates significantly activated and inhibited pathways in primary GC over OM, respectively. (C) Differential expressed immune‐related genes (DEIRGs) between DEGs and IRGs demonstrated by Venn diagram. (D, E) KEGG analysis identified most enriched signaling pathways in the upregulated (D) and downregulated (E) genes of primary GCs when compared to OMs.

A flowchart of the establishment and validation of the ovarian metastasis‐ and immune‐related prognostic signature (OMIRPS) is presented in Figure 1. The FUSCC cohort (*n* = 4), TCGA cohort (*n* = 544), and GSE84437 cohort (*n* = 433) were used as the discovery dataset, training dataset, and validation dataset, respectively. Thus, a total of 981 GC patients were included in the analysis. Transcriptome sequencing of four pairs of primary GCs and matching OMs from FUSCC cohort were performed. Differential expression analysis in the FUSCC cohort identified 1088 differentially expressed genes (DEGs) between primary and ovarian metastatic lesions (|log2FC| > 1, FDR < 0.05), among which 609 genes were upregulated and 479 genes were downregulated in primary GC when compared to OM (Figure 2A). To characterize the biological function and activated signaling pathways underlying DEGs, we conducted both Gene Ontology (GO) analysis of biological process and Ingenuity Pathway Analysis (IPA). The GO analysis revealed that T‐cell activation and lymphocyte differentiation were the most enriched signatures in DEGs (Table S1). The IPA unveiled the significant activation of immune‐related signaling such as crosstalk between dendritic cells and natural killer cells, natural killer cell signaling, Th1, and Th2 Pathway, and PD‐1/PD‐L1 cancer immunotherapy pathway in the comparison between primary GC and OM (Figure 2B). These analyses strongly indicated the significance of immune‐related factors such as tumor immune microenvironment (TIME) in the ovarian metastasis of GC.

Consequently, we focused on deciphering the roles of immune‐related factors in this specific type of distant metastasis. First, 1794 and 1226 immune‐related genes (IRGs) were downloaded from the ImmPort and InnateDB databases, respectively (Table S2). By combining those two IRG lists and removing the overlapped 711 genes, we obtained a total of 2660 IRGs. Then, 182 differentially expressed immune‐related genes (DEIRGs) were identified by intersecting the DEGs and IRGs (Figure 2C), among which 44 DEIRGs were upregulated whereas the other 138 DEIRGs were downregulated in GC samples in comparison with normal samples. Next, we conducted a KEGG pathway analysis to further gain insight into the enriched functional pathways of DEIRGs. Using a threshold of FDR < 0.05, we identified multiple pathways which were enriched in dysregulated DEIRGs. It was revealed that upregulated DEIRGs were significantly involved in immune‐related pathways such as natural killer cell‐mediated cytotoxicity, primary immunodeficiency, T‐cell and B‐cell receptor signaling pathway whereas downregulated DEIRGs were mainly associated with metabolism‐related pathways such as glycosaminoglycan biosynthesis and fatty acid metabolism. The top 10 KEGG pathways of upregulated and downregulated genes were demonstrated as Circos plots in Figure 2D,E, respectively.

### 3.2 Construction and validation of the ovarian metastasis‐ and immune‐related prognostic signature (OMIRPS)

**FIGURE 3:**
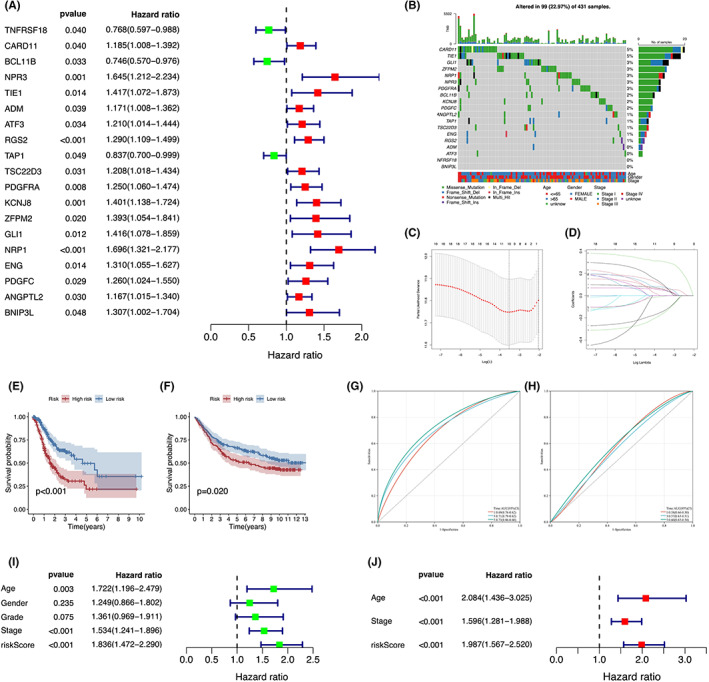
Construction and evaluation of the prognostic value of OMIRPS. (A) Univariate Cox analysis of 19 immune‐related prognostic hub genes from DEIRGs. (B) Profiles of mutation status of 19 immune‐related prognostic hub genes. (C, D) Construction of OMIRPS by LASSO regression analysis. (E, F) Kaplan–Meier survival analysis of OMIRPS‐high and ‐low subgroup in the TCGA cohort (E) and GEO cohort (F). (G, H) Time‐dependent ROC analysis of OMIRPS‐high and ‐low subgroup in the TCGA cohort (G) and GEO cohort (H). (I, J) Univariate and multivariate Cox analysis of clinicopathological factors and the OMIRPS to identify independent prognostic factors.

To obtain immune‐related prognostic hub genes that were significantly involved in OM, we initially conducted Kaplan–Meier survival analysis and univariate Cox regression analysis to evaluate the prognostic significance of DEIRGs. The Kaplan–Meier analysis showed that the expression levels of 19 genes were significantly correlated with the survival of GC patients (Figure S1). Among them, 16 genes (*CARD11*, *NPR3*, *TIE1*, *ADM*, *ATF3*, *RGS2*, *TSC22D3*, *PDGFRA*, *KCNJ8*, *ZFPM2*, *GLI1*, *NRP1*, *ENG*, *PDGFC*, *ANGPTL2*, and *BNIP3L*) were recognized as unfavorable prognostic genes with hazard ratios (HRs) >1, while three genes (*TNFRSF18*, *BCL11B*, and *TAP1*) were deemed as favorable prognostic genes with HRs <1 (Figure 3A). The somatic mutations and copy number variations of these 19 genes were also investigated. As shown in Figure 3B, missense mutations were the most common mutation type, and *CARD11* and *TIE1* exhibited the highest mutation frequency (5%) followed by *GLI1* (3%), *ZFPM2* (3%), *NRP1*(3%), *NPR3* (3%), and *PDGFRA* (3%).

Next, the 19 immune‐related prognostic hub genes identified in the TCGA training set were submitted for LASSO regression analysis to construct the ovarian metastasis‐ and immune‐related and prognostic signature (OMIRPS) (Figure 3C,D). The coefficient of each gene was calculated, followed by the establishment of a signature which consists of six immune‐related genes (*TNFRSF18*, *CARD11*, *BCL11B*, *NRP1*, *BNIP3L*, and *ATF3*). The OMIRPS score = *TNFRSF18* × (−0.25) + *CARD11* × 0.21 + *BCL11B* × (−0.37) + *NRP1* × 0.42 + *BNIP3L* × 0.29 + *ATF3* × 0.15. The patients were divided into OMIRPS‐low (*n* = 182) and OMIRPS‐high (*n* = 180) subgroups according to the median risk score.

The prognostic value of OMIRPS was validated by using the TCGA cohort and GSE84437 cohort. First, the Kaplan–Meier survival analysis revealed that GC patients in the OMIRPS‐high subgroup had a significantly worse overall survival (OS) than those who were in the OMIRPS‐low subgroup for both the TCGA (*p <* 0.001) (Figure 3E) and GSE84437 (*p =* 0.020) (Figure 3F) cohorts. Next, the time‐dependent ROC analysis showed that the AUC values of OMIRPS for predicting OS in the TCGA cohort were 0.69, 0.71, and 0.73 at 1, 3, and 5 years, respectively, suggesting a good predictive accuracy (Figure 3G). Consistent with this result, AUC values in the GSE84437 cohort were 0.58, 0.657, and 0.60 at 1, 3, and 5 years, respectively (Figure 3H). Furthermore, both univariate and multivariate Cox regression analyses were conducted in the TCGA cohort to investigate if OMIRPS could serve as an independent prognostic factor of GC patients. The univariate Cox regression analysis indicated that age, TNM stage, and OMIRPS were significantly associated with the OS of patients. The multivariate Cox regression analysis confirmed that OMIRPS remained an independent prognostic factor (Figure 3I,J). Accordingly, OMIRPS‐low and ‐high subgroups were defined as low‐ and high‐risk subgroups, respectively.

### 3.3 Molecular characteristics of OMIRPS‐high and ‐low subgroup

**FIGURE 4:**
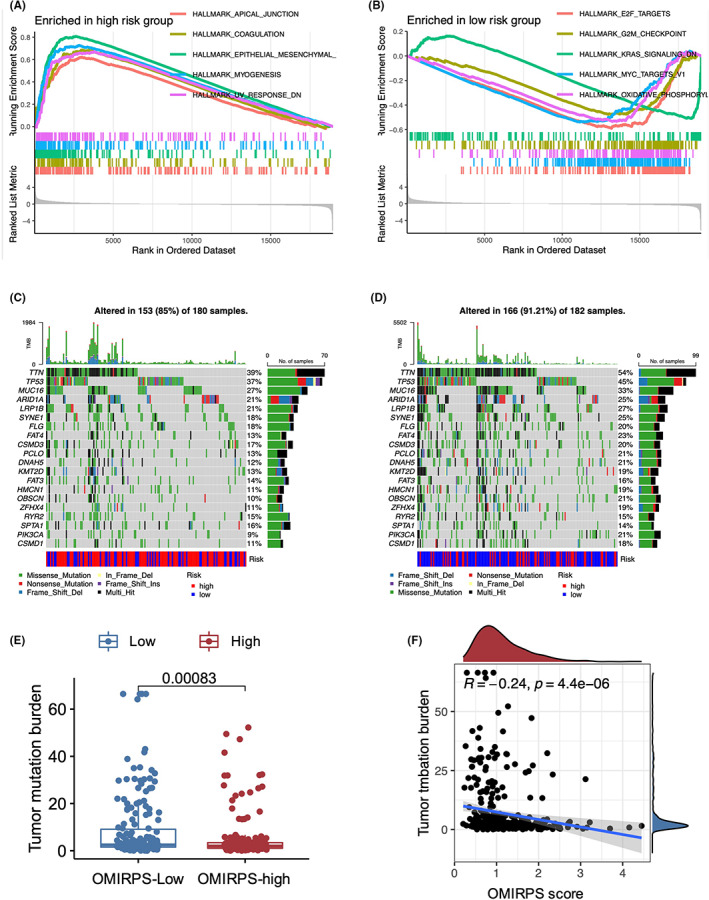
Molecular characteristics of different OMIRPS subgroups. (A, B) Enriched gene sets in OMIRPS‐high (A) and OMIRPS‐low (B) subgroup (*p* < 0.05, FDR < 0.25) revealed by GSEA analysis. (C, D). Significantly mutated genes in the mutated samples of OMIRPS‐high (C) and OMIRPS‐low (D) subgroup demonstrated by waterfall plots. The names of top mutated genes and their corresponding mutation rate were listed on the left and right side whereas the total number of mutations and the mutation types were listed on the top and bottom, respectively. Different mutation types were represented by various colors. (E) Differential tumor mutation burden (TMB) between OMIRPS‐high and ‐low subgroup analyzed by Wilcoxon rank‐sum tests. (F) Correlation between TMB and OMIRPS score analyzed by Pearson correlation analysis.

To gain insight into the biological features of different OMIRPS subgroups, we performed a Gene Set Enrichment Analysis (GSEA) regarding Hallmark signaling pathways in the TCGA cohort. The genes of the OMIRPS‐high samples were mainly enriched in cancer‐related pathways such as epithelial–mesenchymal transition (EMT). Considering the critical role of aberrant CDH1, the protein coding gene of E‐cadherin, in both the EMT process and hereditary diffuse GC progression, we compared the CDH1 expression between the OMIRPS‐high and‐low subgroup and did not identify any significant differences. Additionally, the OMIRPS score between CDH1 mutant and wild‐type GC patients were also similar (Figure S2). On the other hand, the genes of the OMIRPS‐low samples were enriched in cell cycle and metabolism‐related pathways, such as E2F targets, G2M checkpoint, and Oxidative phosphorylation pathways. The top five enriched signaling pathways in the OMIRPS‐low and OMIRPS‐high subgroups are displayed in Figure 4A,B, respectively. Detailed GSEA results are listed in Table S3.

Somatic mutations in different OMIRPS subgroups were also investigated. As shown in Figure 4C,D, *TTN*, *TP53*, *MUC16*, *ARID1A*, and *LRP1B* were the most commonly mutated genes in both subgroups. Notably, the overall mutation rate was significantly higher in the OMIRPS‐low subgroup compared to the OMIRPS‐high subgroup (91.21% vs. 85.00%, *p* < 0.001). Mutation of each of the top 20 genes was more common in the OMIRPS‐low subgroup than in the OMIRPS‐high subgroup. Next, we investigated the correlation between OMIRPS score and tumor mutation burden (TMB). It was demonstrated that TMB was significantly higher in the OMIRPS‐low subgroup than in the OMIRPS‐high subgroup (*p* < 0.001, Figure 4E), and TMB was negatively correlated with the OMIRPS score (*r* = −0.24, *p* < 0.001, Figure 4F).

### 3.4 Immune characteristics of OMIRPS‐high and ‐low subgroup

**FIGURE 5:**
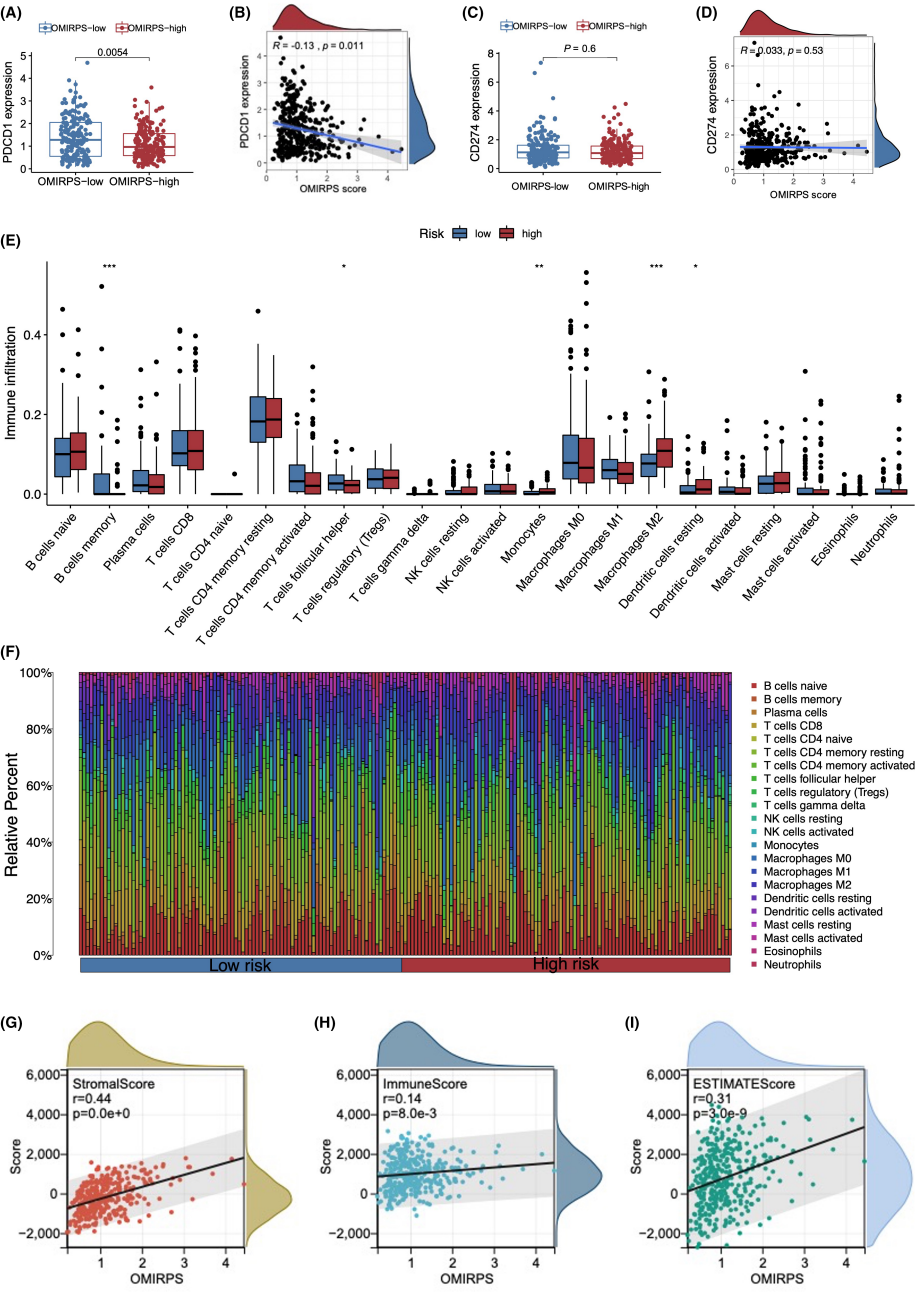
Immune characteristics of different OMIRPS subgroups. (A) Differential expression of PDCD‐1 between OMIRPS‐high and ‐low subgroup analyzed by Wilcoxon rank‐sum tests. (B) Correlation between PDCD‐1 expression and OMIRPS score analyzed by Pearson correlation analysis. (C) Comparable expression of CD274 between OMIRPS‐high and ‐low subgroup analyzed by Wilcoxon rank‐sum tests. (D) Correlation between CD274 expression and OMIRPS score analyzed by Pearson correlation analysis. (E) CIBERSORT analyses uncovered the proportions of tumor microenvironment cells in OMIRPS‐high and ‐low subgroup. The immune score of different subgroups was represented by the scattered dots, with the median value and interquartile range represented by the thick lines and bottom and top of the boxes, respectively. (F) Heatmap demonstrating the proportions of 22 different TME cells for patients in OMIRPS‐high and ‐low subgroup, respectively. (G–I) ESTIMATE and OMIRPS score in TCGA‐STAD cohort. (G) Significant correlation between StromalScore and OMIRPS score, (H) Insignificant correlation between ImmuneScore and OMIRPS score and (I) Correlation between overall ESTIMATEScore and OMIRPS score.

Immune checkpoints represent an immunosuppressive mechanism that allows cancer cells to escape antitumor immunity. Key immune checkpoint molecules such as PD‐1 (encoded by *PDCD1*), PD‐L1 (encoded by *CD274*), CTLA4, and LAG3 have been identified as potential immunotherapeutic targets. We analyzed the association between the OMIRPS score and the expression of immune checkpoint molecules. It was shown that PDCD‐1 but not CD274 was significantly overexpressed in OMIRPS‐low subgroup compared to the OMIRPS‐high subgroup and the expression level of PD‐1 was negatively correlated with the OMIRPS score (Figure 5A–D). Alternatively, the expression levels of CTLA4 and LAG3 were similar between the two subgroups (Figure S3). In addition, as multiple immune regulatory molecules such as IFN‐γ and TGF‐β play essential roles in the immune microenvironment, we compared the expression levels of IFNG, TGFB1, and TGFB2 between the OMIRPS‐high and‐low groups. It was demonstrated that the TGFB1 and TGFB2 expressions were significantly higher in the OMIRPS‐high group, while the IFNG expression was significantly higher in OMIRPS‐low group (Figure S4).

To gain further insights into the correlation between infiltrating immune cells and OMIRPS subgroups, we compared the distribution of 22 types of immune cells in different subgroups using the CIBERSORT algorithm. The landscape of immune cells distribution in each sample within the TCGA cohort is illustrated in Figure 5E. The analysis revealed that memory B cells and follicular helper‐T cells were more abundant in the OMIRPS‐low subgroup, suggesting a robust antitumor immune response. In contrast, monocytes, M2 macrophages, and resting dendritic cells were more enriched in the OMIRPS‐high subgroup (Figure 5F), indicating its immunosuppressive status and poor prognosis. In addition to this, we evaluated the prognostic significance of 22 immune cells in the TCGA cohort. It was found that higher infiltration levels of resting dendritic cells, neutrophils, and M2 Macrophages were significantly associated with worse prognoses, while higher infiltration levels of M0 Macrophages, resting Mast cells, CD8 T‐cells, and CD4 memory T‐cells activated were markedly correlated with favorable survival outcomes (Figure S5). Furthermore, it was noted that in addition to immune cells, stromal components in the tumor immune microenvironment may also contribute to both tumor immunity and progression. Therefore, we evaluated the ESTIMATE score which consists of both the stromal and immune score in the TCGA‐STAD cohort with the application of an ESTIMATE algorithm. Our follow‐up analysis showed that OMIRPS was significantly correlated with the stromal score (*R* = 0.44) but not with the immune score (*R* = 0.14) (Figure 5G–I), indicating the significance of active interaction between tumor, stroma, and immune cells in GC ovarian metastasis.

### 3.5 Predicting immunotherapy efficacy and immune escape in GC patients by OMIRPS

**FIGURE 6:**
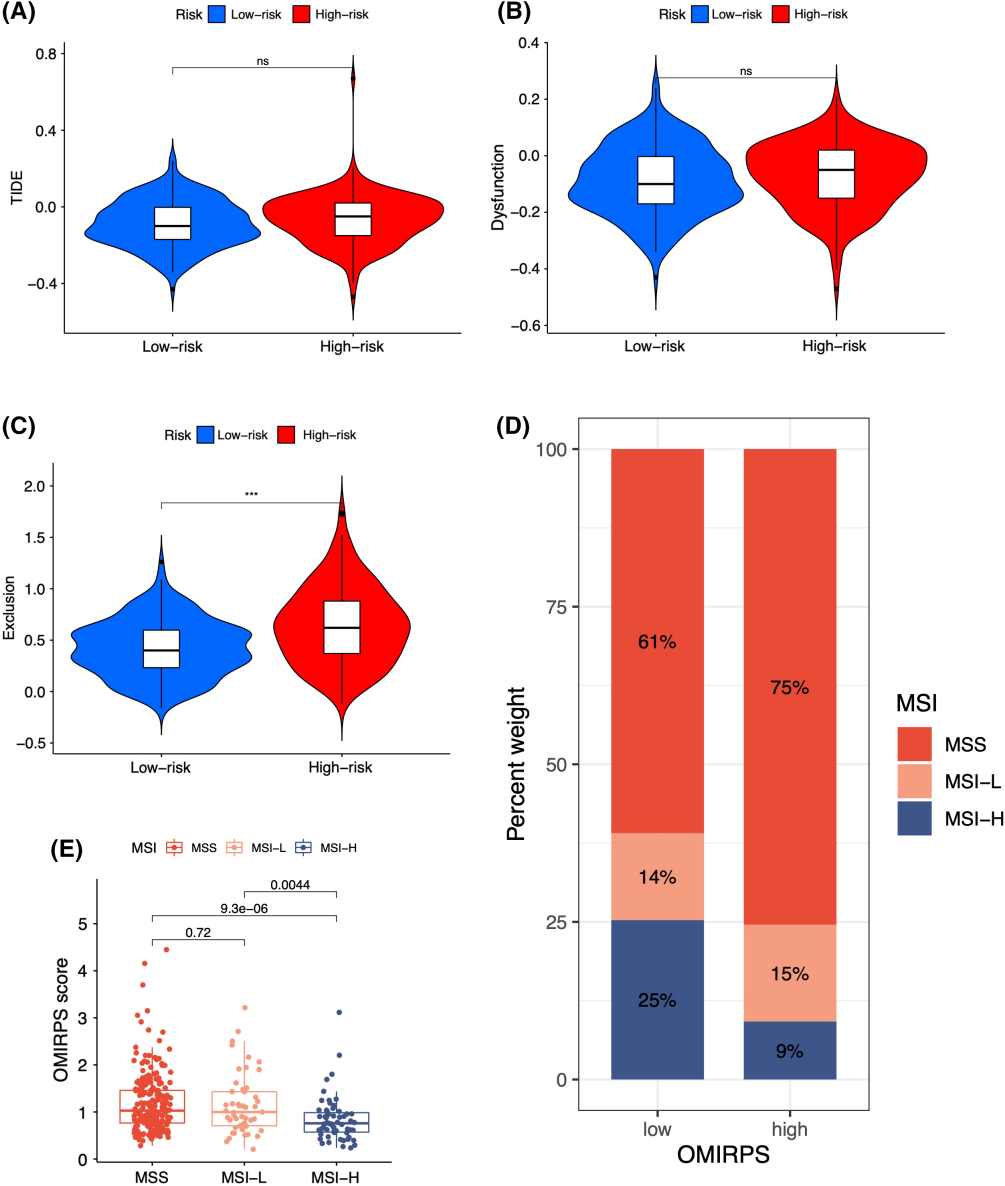
The predictive value of OMIRPS in patients receiving immunotherapy response. (A, B) Comparable TIDE and dysfunction score between OMIRPS‐high and ‐low subgroup. (C) Differential exclusion score between OMIRPS‐high and ‐low subgroups. (D) Differential MSI status between OMIRPS‐high and ‐low subgroup. (E) Differential OMIRPS score between subgroups of patients with various microsatellite status.

The Tumor Immune Dysfunction and Exclusion (TIDE) online tool (http://tide.dfci.harvard.edu/) was used to evaluate the potential benefit from immunotherapy efficacy by assessing the TIDE score, immune dysfunction score, immune exclusion score, and MSI status in the TCGA cohort. The TIDE score was positively correlated with immune escape, resulting in less benefit from immunotherapy, and an unfavorable survival outcome. Although TIDE score and immune dysfunction score were comparable between OMIRPS‐high and ‐low subgroups (Figure 6A,B), the immune exclusion score was significantly higher in OMIRPS‐high subgroup than in the OMIRPS‐low subgroup (*p* < 0.05) (Figure 6C), implying that GC patients in OMIRPS‐high subgroup had worse prognosis and might benefit less from immunotherapy than OMIRPS‐low patients. Meanwhile, we compared OMIRPS with the MSI status in TCGA cohort. It was demonstrated that the proportion of MSI‐high in the OMIRPS‐low group was significantly higher than that in the OMIRPS‐high group (Figure 6D) whereas the OMIRPS score of the MSI‐high group was significantly lower than those of the MSI‐low and Microsatellite Stable (MSS) groups (Figure 6E). Considering the remarkably improved immunotherapy response in MSI‐high tumor, these results indicated the potential value of OMIRPS for the prediction of immunotherapy efficacy.

### 3.6 Comparison between OMIRPS grouping and other classifications

**FIGURE 7:**
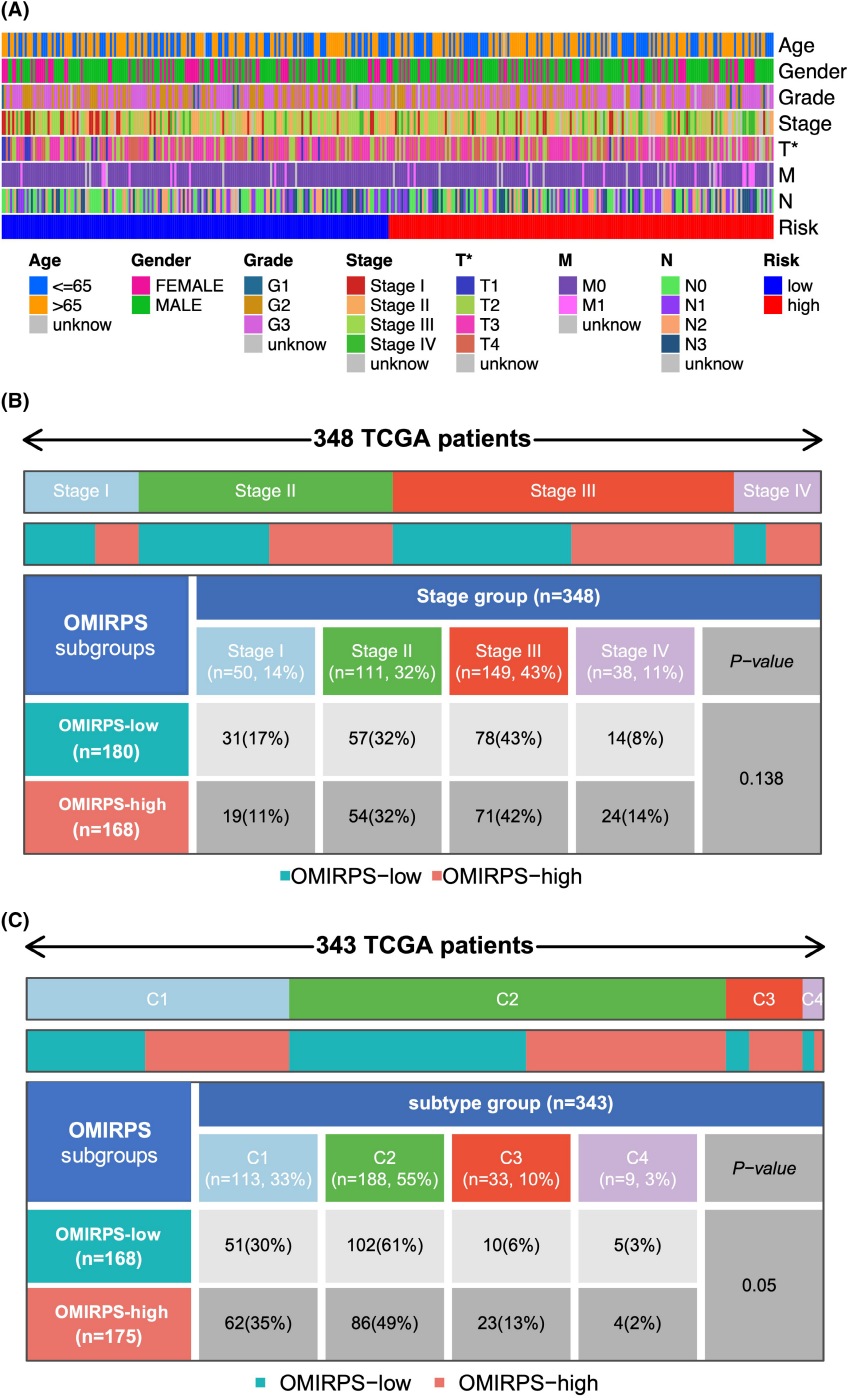
Distribution of clinicopathological parameters and Immune Subtype (IS) in different OMIRPS subgroups. (A) Distribution of clinicopathological parameters including age, gender, tumor grade, pathological T, N, and M stage in OMIRPS‐high and ‐low subgroup, respectively. Map. (B) Distribution of pathological stages (I to IV) in OMIRPS‐high and ‐low subgroup, respectively. (C) Distribution of Immune Subtypes (IS) (C1 to C4) in OMIRPS‐high and ‐low subgroup, respectively. Data are shown in the form of heatmap.

Next, we compared the clinicopathologic characteristics of patients between OMIRPS‐low and OMIRPS‐high subgroups in TCGA cohort. It was noted that high‐risk group tended to have higher proportion of advanced pathological T stage (*p* < 0.05) whereas the rest of the clinicopathologic characteristics were comparable between the two subgroups (Figure 7A). We further analyzed the relationship between OMIRPS grouping and TNM classification and found no correlation between them (*p* = 0.138, chi‐square test) (Figure 7B), indicating that the prognostic value of OMIRPS scoring was independent of pathological classification.

On the other hand, OMIRPS grouping was compared to Immune Subtype (IS),^14^ which was characterized by a distinct distribution of scores over the five immune expression signatures: C1 (Wound Healing), C2 (IFN‐γ Dominant), C3 (Inflammatory), C4 (Lymphocyte Depleted), C5 (Immunologically Quiet), and C6 (TGF‐β Dominant). As shown in Figure 7C, the OMIRPS‐high group had more IS‐C1 and IS‐C3, while IS‐C2 and IS‐C4 were more likely to be enriched in OMIRPS‐low group. Statistical analysis showed that OMIRPS grouping was associated with Immune Subtypes with borderline significance (*p* = 0.05).

## 4 DISCUSSION

Ovarian metastasis considerably threatens the clinical outcome and contributes to treatment failure of female GC patients.^3^ Although mounting evidence showed the effectiveness of immunotherapy in GC,^9^, ^17^, ^18^ it has not been extensively applied to the treatment of patients with OM yet. Moreover, only a limited number of patients can benefit from ICI due to the low overall response rate.^10^ These findings highlight the urgent need to construct validated signatures to identify patients who could be responsive to and benefit from immunotherapy. Unlike most gene signatures derived from analyses of TCGA, GEO, and other public databases,^19^, ^20^, ^21^, ^22^ we utilized transcriptome profiling data of paired primary and ovarian metastatic lesions from our own FUSCC cohort and integrated it with multiple public databases to develop a predictive biomarker. To our knowledge, this is the first OM‐related gene signature to predict the prognosis and immunotherapy efficacy for GC patients.

By intersecting DEGs between GC and OM from the FUSCC cohort and IRGs from the ImmPort and InnateDB, we identified 19 immune‐related hub genes and constructed OMIRPS based on six genes (*TNFRSF18*, *CARD11*, *BCL11B*, *NRP1*, *BNIP3L*, and *ATF3*), among which *TNFRSF18* and *BCL11B* was positively associated with patient OS while the rest were the opposite. TNFRSF18, also known as the glucocorticoid‐induced tumor necrosis factor receptor‐related protein (GITR), was highly expressed in activated T cells and regulatory T cells (Tregs).^23^ TNFRSF18 has emerged as a novel immunotherapy target as it induces a robust proliferation of T effector cells and hampers the suppression of Tregs.^24^ Studies have demonstrated the immune effects and mechanistic evidence of GITR agonism‐based T‐cell reinvigoration combined with ICI in the treatment of solid tumors.^25^, ^26^ As for BCL11B, a C2H2 zinc finger transcription factor and a haploinsufficient tumor suppressor, it exerts inhibitory effects on tumor progression through a wide range of mechanisms.^27^, ^28^, ^29^ Notably, it is required for the development and activation of mature T cells and participates in the development of invariant natural killer T (iNKT) cells, which play a key role in immune regulation and antitumor responses.^30^ Mechanism studies revealed that BCL11B prevents cancer immune evasion by acting as a competitive endogenous RNA to upregulate MICA and MICB, which essentially control tumor immune surveillance.^31^ On the other hand, the remaining four immune‐related genes are reportedly oncogenic. For instance, CARD11, also known as CARMA1, is mainly expressed in lymphoid tissues and is involved in both adaptive immunity and carcinogenesis.^32^ Disruption of the CARMA1–BCL10–MALT1 (CBM) signalosome complex by genetic deletion of CARMA1 resulted in antitumor effects through the loss of suppressive function and the gain of effector activity by Tregs.^33^ CARMA1 deletion combined with ICI improved the patient response to anti‐PD‐1 monotherapy and induced tumor suppression.^33^ NRP1 intrinsically regulates both Treg cell and CD8+ T‐cell functions to collectively impede antitumor immunity in the tumor microenvironment.^34^ It also contributes to tumor angiogenesis by acting as a co‐receptor with VEGFR as a recent study demonstrated that anti‐NRP1 monoclonal antibody (MNRP1685A) inhibited the VEGF pathway in solid tumors.^35^ ATF3 is an environmental stress‐induced transcription factor. It regulates immunity and oncogenesis through transcriptional activation or repression and reciprocally functions as an oncogene or tumor suppressor in several malignancies.^36^ In GC, ATF3 was found to be upregulated by EBV infections, whereas the downregulation of ATF3 suppressed the proliferation of EBV‐infected gastric cells.^37^ In contrast, Huang et al. showed that ATF3 overexpression inhibited cell invasiveness and decreased stemness and EMT‐promoting genes, indicating a suppressive role of ATF3 in tumor progression.^38^ BNIP3L, a member of the BCL‐2 family, is reportedly induced by p53 under hypoxia and is critically involved in p53‐mediated autophagy.^39^ It serves as a predictive marker for both prognosis and response to angiogenesis inhibitors in GC.^40^, ^41^ In summary, the construction of OMIRPS was based on a collection of immune‐related oncogenic and/or tumor suppressor genes which originated from the gene expression and mutation data of FUSCC and public databases.

To characterize the molecular features underlying different OMIRPS subgroups, we studied the functionally enriched signaling pathways and gene mutations of each group. In line with previous reports, GSEA analyses revealed that genes in different subgroups were enriched in distinctive sets.^42^, ^43^, ^44^ For instance, TGF‐β signaling and EMT pathways were enriched in the OMIRPS‐high subgroup, which partially explained its poor prognosis, especially considering that TGF‐β promotes cancer progression by pressing cancer cells into EMT which results in metastasis and chemotherapy resistance.^45^ In the OMIRPS‐low subgroup, however, cell cycle‐related pathways such as E2F targets pathway were enriched. It was reported that the E2F expression is correlated with tumor suppression and immune infiltration in GC.^46^, ^47^ Apart from functional enrichment analyses, we also conducted somatic mutation studies of OMIRPS subgroups. Although the top mutated genes such as *TTN*, *TP53*, *MUC16*, *ARID1A*, and *LRP1B* were identical between the two subgroups, the tumor mutation burden (TMB) was significantly higher in the OMIRPS‐low subgroup and the TMB was negatively correlated with the OMIRPS score. Notably, mutations of *TTN* and *MUC16* were associated with the TMB and could predict the immunotherapy efficacy in GC and pan‐cancer.^48^ TMB, the number of non‐synonymous single nucleotide variants (nsSNVs) in a tumor, affects the odds of producing immunogenic peptides and thereby influences T‐cell‐mediated antitumor activity and the patient's response to ICI.^49^, ^50^ Consequently, TMB is proposed as a key biomarker to predict both the efficacy and prognosis of patients receiving immunotherapy in multiple malignancies such as lung, colon, and gastric cancer,^51^, ^52^, ^53^ which is consistent with our results demonstrating that TMB is different between OMIRPS subgroups. Therefore, our molecular characterization shed light on the mechanisms underlying the survival difference between different OMIRPS subgroups.

In addition to TMB, expressions of immune checkpoint molecules such as PD‐1 and PD‐L1 are also recognized as key biomarkers of immunotherapy efficacy.^50^ Our analyses demonstrated a significant increase of PD‐1, the target of monoclonal antibody (pembrolizumab) approved for late‐stage GC and a marker of tumor‐reactive T cells and enhanced T‐cell receptor signaling,^54^ in the OMIRPS‐low subgroup. In fact, PD‐1 expression was correlated with CD8+ T‐cell density at the invasion front of the microsatellite unstable GC tissues,^55^ whereas TCGA‐based profiling of PD gene expression showed the significant association of PD‐1 expression with the improved prognosis of GC patients.^56^ As for PD‐L1, although emerging data suggests that patients with its overexpression have improved clinical outcomes with anti‐PD‐1 therapy,^57^ we observed a similar expression value of PD‐L1 between subgroups. Nevertheless, IHC‐based detection of PD‐L1 expression was more widely accepted than transcriptomics‐based measurements.^58^ Therefore, the relationship between PD‐L1 expression and OMIRPS requires further clarification. Furthermore, we also introduced the TIDE score to evaluate whether OMIRPS could discriminate between responders and non‐responders to immunotherapy. The algorithm analyzed key factors underlying the two primary mechanisms of tumor immune escape including high infiltration of cytotoxic T lymphocytes (CTL)‐induced T‐cell dysfunction and low infiltration of CTL‐induced T‐cell exclusion.^59^ In fact, TIDE outperforms known immunotherapy biomarkers such as the expression of TMB and PD‐L1 in solid tumors such as melanoma and lung cancer.^60^ In our study, OMIRPS was correlated with the immune exclusion score, which indicated that OMIRPS‐high patients may be less responsive to immunotherapy. Additionally, the proportion of MSI‐high was significantly lower in the OMIRPS‐high subgroup. Considering the positive correlation between MSI and increased TMB and enhanced response to ICI,^61^, ^62^ it is assumed that low responsiveness of the OMIRPS‐high subgroup is partially attributed to less lymphocyte infiltration caused by the reduced neoantigen from tumor mutation.

In the meantime, we characterized the immunological nature of the OMIRPS subgroups by profiling their distinctive composition of infiltrating immune cells. CIBERSORT is a computational algorithm used to enumerate cell fractions from bulk gene expression profiles. It has been extensively applied in profiling the composition of tumor infiltrating immune cells in a wide range of tumors.^63^ Accordingly, follicular helper‐T cells and memory‐B cells were more enriched in the OMIRPS‐low subgroup whereas monocytes, M2 macrophages, and resting dendritic cells were more enriched in the OMIRPS‐high subgroup. Among them, follicular helper‐T (Tfh) cells, a subset of CD4+ T cells, facilitate B cell‐mediated antibody response and is often correlated with improved survival of several solid tumors.^64^ In GC, Tfh cells suppress tumor progression by secreting diverse cytokines and antibodies to promote tumor‐associated lymphocytes.^65^ In contrast, chronic inflammation‐related M2 macrophages have been repeatedly shown to favor invasive malignant phenotype and confers poor prognosis of breast, lung, ovarian, prostate, gastric cancer etc..^66^, ^67^, ^68^, ^69^ As we also compared OMIRPS with Immune Subtype (IS) classification based on distinctive immune expression signatures,^14^ there were more C1 (Wound Healing) and C3 (Inflammatory) in the OMIRPS‐high group, while there were more C2 (IFN‐γ Dominant) and C4 (Lymphocyte Depleted) in the OMIRPS‐low group. These results demonstrated the correlation between different types of immune cells and signatures with the distinctive prognosis of GC patients and reinforced our survival analysis.

Considering the limitations of the present study, cautions must be exercised when interpreting and utilizing the predictive signature. Although we aimed to develop an OM‐related predictive signature, molecular investigations of OM are so rare that publicized databases which can ideally match our investigative scope remains null. Instead, we used the TCGA‐STAD database as the training cohort and GSE84437, a representative microarray data of Asian population from Yonsei gastric cancer cohort, as the validation cohort. Consequently, the effectiveness and validity of OMIRPS in the application of GC patients with OM have not been rigidly tested yet. Moreover, we used retrospective datasets to construct OMIRPS predicting both the prognosis and immunotherapy efficacy of patients. Therefore, validation of our signature in a prospective clinical trial with sufficient patient number is inevitably necessary. Additionally, only four pairs of primary and ovarian metastatic tumors were submitted for RNA‐seq to identify key candidate genes regulating OM. The small sample size in our study limits the broad reflection of molecular landscape underlying OM, so more paired specimens are needed for further investigation.

In conclusion, transcriptome profiling of paired primary and ovarian metastatic lesions revealed the significance of tumor immune microenvironment and other immune‐related factors in OM. Based on DEGs from RNA‐seq analysis and IRGs from immune‐related databases, we constructed OMIRPS, an OM‐related prognostic signature which distinguishes the molecular features and immune characteristics between GC patients. OMIRPS serves as a robust biomarker to predict the prognosis of patients and their potential response to immunotherapy, which requires additional studies to validate in the future.

## AUTHOR CONTRIBUTIONS

Conception and design: Jianpeng Gao, Hongda Pan, and Xiaowen Liu. Administrative support: Hongda Pan and Xiaowen Liu. Provision of study materials or patients: Jianpeng Gao and Hongda Pan. Collection and assembly of data: Jianpeng Gao, Shiying Huo, Yu Zhang, and Hongda Pan. Data analysis and interpretation: Jianpeng Gao, Shiying Huo, Yu Zhang, Zhenxiong Zhao, Hongda Pan, and Xiaowen Liu. Manuscript writing: All authors. Final approval of manuscript: All authors.

## CONFLICT OF INTEREST

The authors declare that they have no conflict of interest.

## ETHICAL APPROVAL STATEMENT

The study was approved by the ethics committee of the Fudan University Shanghai Cancer Center and signed informed consent was obtained from each patient.

## PATIENT CONSENT STATEMENT

Informed consent was obtained from all participants included in this study.

## Supporting information


Figure S1

Figure S2

Figure S3

Figure S4

Figure S5

Table S1–S3

## Data Availability

The datasets used for the current study are available from the corresponding authors upon reasonable request.

## References

[1] Van Cutsem E , Sagaert X , Topal B , Haustermans K , Prenen H . Gastric cancer. Lancet. 2016;388(10060):2654‐2664. 27156933 10.1016/S0140-6736(16)30354-3

[2] Wang J , Shi YK , Wu LY , et al. Prognostic factors for ovarian metastases from primary gastric cancer. Int J Gynecol Cancer. 2008;18(4):825‐832. 17892453 10.1111/j.1525-1438.2007.01078.x

[3] Kubeček O , Laco J , Špaček J , et al. The pathogenesis, diagnosis, and management of metastatic tumors to the ovary: a comprehensive review. Clin Exp Metastasis. 2017;34(5):295‐307. 28730323 10.1007/s10585-017-9856-8 PMC5561159

[4] Yan D , Du Y , Dai G , Huang L , Xu Q , Yu P . Management of synchronous Krukenberg tumors from gastric cancer: a single‐center experience. J Cancer. 2018;9(22):4197‐4203. 30519320 10.7150/jca.25593 PMC6277623

[5] Yu P , Huang L , Cheng G , et al. Treatment strategy and prognostic factors for Krukenberg tumors of gastric origin: report of a 10‐year single‐center experience from China. Oncotarget. 2017;8(47):82558‐82570. 29137284 10.18632/oncotarget.19759 PMC5669910

[6] Waldman AD , Fritz JM , Lenardo MJ . A guide to cancer immunotherapy: from T cell basic science to clinical practice. Nat Rev Immunol. 2020;20(11):651‐668. 32433532 10.1038/s41577-020-0306-5 PMC7238960

[7] Zhang JY , Yan YY , Li JJ , Adhikari R , Fu LW . PD‐1/PD‐L1 based combinational cancer therapy: icing on the cake. Front Pharmacol. 2020;11:722. 32528284 10.3389/fphar.2020.00722 PMC7247431

[8] Lin EM , Gong J , Klempner SJ , Chao J . Advances in immuno‐oncology biomarkers for gastroesophageal cancer: programmed death ligand 1, microsatellite instability, and beyond. World J Gastroenterol. 2018;24(25):2686‐2697. 29991874 10.3748/wjg.v24.i25.2686 PMC6034145

[9] Janjigian YY , Shitara K , Moehler M , et al. First‐line nivolumab plus chemotherapy versus chemotherapy alone for advanced gastric, gastro‐oesophageal junction, and oesophageal adenocarcinoma (CheckMate 649): a randomised, open‐label, phase 3 trial. Lancet. 2021;398(10294):27‐40. 34102137 10.1016/S0140-6736(21)00797-2 PMC8436782

[10] Baxter MA , Middleton F , Cagney HP , Petty RD . Resistance to immune checkpoint inhibitors in advanced gastro‐oesophageal cancers. Br J Cancer. 2021;125(8):1068‐1079. 34230609 10.1038/s41416-021-01425-7 PMC8505606

[11] Nishino M , Ramaiya NH , Hatabu H , Hodi FS . Monitoring immune‐checkpoint blockade: response evaluation and biomarker development. Nat Rev Clin Oncol. 2017;14(11):655‐668. 28653677 10.1038/nrclinonc.2017.88 PMC5650537

[12] Zeng D , Li M , Zhou R , et al. Tumor microenvironment characterization in gastric cancer identifies prognostic and Immunotherapeutically relevant gene signatures. Cancer Immunol Res. 2019;7(5):737‐750. 30842092 10.1158/2326-6066.CIR-18-0436

[13] Wan L , Tan N , Zhang N , Xie X . Establishment of an immune microenvironment‐based prognostic predictive model for gastric cancer. Life Sci. 2020;261:118402. 32926930 10.1016/j.lfs.2020.118402

[14] Thorsson V , Gibbs DL , Brown SD , et al. The immune landscape of cancer. Immunity. 2018;48(4):812‐30.e14. 29628290 10.1016/j.immuni.2018.03.023 PMC5982584

[15] Bhattacharya S , Dunn P , Thomas CG , et al. ImmPort, toward repurposing of open access immunological assay data for translational and clinical research. Sci Data. 2018;5:180015. 29485622 10.1038/sdata.2018.15 PMC5827693

[16] Breuer K , Foroushani AK , Laird MR , et al. InnateDB: systems biology of innate immunity and beyond‐‐recent updates and continuing curation. Nucleic Acids Res. 2013;41(Database issue):D1228‐D1233. 23180781 10.1093/nar/gks1147 PMC3531080

[17] Kang YK , Boku N , Satoh T , et al. Nivolumab in patients with advanced gastric or gastro‐oesophageal junction cancer refractory to, or intolerant of, at least two previous chemotherapy regimens (ONO‐4538‐12, ATTRACTION‐2): a randomised, double‐blind, placebo‐controlled, phase 3 trial. Lancet. 2017;390(10111):2461‐2471. 28993052 10.1016/S0140-6736(17)31827-5

[18] Fuchs CS , Doi T , Jang RW , et al. Safety and efficacy of pembrolizumab monotherapy in patients with previously treated advanced gastric and gastroesophageal junction cancer: phase 2 clinical KEYNOTE‐059 trial. JAMA Oncol. 2018;4(5):e180013. 29543932 10.1001/jamaoncol.2018.0013 PMC5885175

[19] Kim JC , Heo YJ , Kang SY , Lee J , Kim KM . Validation of the combined biomarker for prediction of response to checkpoint inhibitor in patients with advanced cancer. Cancers (Basel). 2021;13(10):2316. 34065963 10.3390/cancers13102316 PMC8151730

[20] Yue T , Zuo S , Zhu J , et al. Two similar signatures for predicting the prognosis and immunotherapy efficacy of stomach adenocarcinoma patients. Front Cell Dev Biol. 2021;9:704242. 34414187 10.3389/fcell.2021.704242 PMC8369372

[21] Noh MG , Yoon Y , Kim G , et al. Practical prediction model of the clinical response to programmed death‐ligand 1 inhibitors in advanced gastric cancer. Exp Mol Med. 2021;53(2):223‐234. 33547412 10.1038/s12276-021-00559-1 PMC8080676

[22] Chen Y , Sun Z , Chen W , et al. The immune subtypes and landscape of gastric cancer and to predict based on the whole‐slide images using deep learning. Front Immunol. 2021;12:685992. 34262565 10.3389/fimmu.2021.685992 PMC8273735

[23] Ronchetti S , Ricci E , Petrillo MG , Cari L , Migliorati G , Nocentini G , Riccardi C Glucocorticoid‐induced tumour necrosis factor receptor‐related protein: a key marker of functional regulatory T cells. J Immunol Res 2015;2015:171520, 1, 17. 25961057 10.1155/2015/171520 PMC4413981

[24] Buzzatti G , Dellepiane C , Del Mastro L . New emerging targets in cancer immunotherapy: the role of GITR. ESMO Open. 2020;4(Suppl 3):e000738. 32817129 10.1136/esmoopen-2020-000738 PMC7451269

[25] Zappasodi R , Sirard C , Li Y , et al. Rational design of anti‐GITR‐based combination immunotherapy. Nat Med. 2019;25(5):759‐766. 31036879 10.1038/s41591-019-0420-8 PMC7457830

[26] Papadopoulos KP , Autio K , Golan T , et al. Phase I study of MK‐4166, an anti‐human glucocorticoid‐induced TNF receptor antibody, alone or with pembrolizumab in advanced solid tumors. Clin Cancer Res. 2021;27(7):1904‐1911. 33355238 10.1158/1078-0432.CCR-20-2886 PMC9094061

[27] Obata M , Kominami R , Mishima Y . BCL11B tumor suppressor inhibits HDM2 expression in a p53‐dependent manner. Cell Signal. 2012;24(5):1047‐1052. 22245141 10.1016/j.cellsig.2011.12.026

[28] Yang WJ , Sun YF , Jin AL , et al. BCL11B suppresses tumor progression and stem cell traits in hepatocellular carcinoma by restoring p53 signaling activity. Cell Death Dis. 2020;11(10):895. 33093445 10.1038/s41419-020-03115-3 PMC7581528

[29] Gutierrez A , Kentsis A , Sanda T , et al. The BCL11B tumor suppressor is mutated across the major molecular subtypes of T‐cell acute lymphoblastic leukemia. Blood. 2011;118(15):4169‐4173. 21878675 10.1182/blood-2010-11-318873 PMC3204734

[30] Albu DI , VanValkenburgh J , Morin N , et al. Transcription factor Bcl11b controls selection of invariant natural killer T‐cells by regulating glycolipid presentation in double‐positive thymocytes. Proc Natl Acad Sci USA. 2011;108(15):6211‐6216. 21444811 10.1073/pnas.1014304108 PMC3076841

[31] Qian M , Geng J , Luo K , et al. BCL11B regulates MICA/B‐mediated immune response by acting as a competitive endogenous RNA. Oncogene. 2020;39(7):1514‐1526. 31673069 10.1038/s41388-019-1083-0

[32] Bedsaul JR , Carter NM , Deibel KE , et al. Mechanisms of regulated and dysregulated CARD11 signaling in adaptive immunity and disease. Front Immunol. 2018;9:2105. 30283447 10.3389/fimmu.2018.02105 PMC6156143

[33] Di Pilato M , Kim EY , Cadilha BL , et al. Targeting the CBM complex causes T(reg) cells to prime tumours for immune checkpoint therapy. Nature. 2019;570(7759):112‐116. 31092922 10.1038/s41586-019-1215-2 PMC6656391

[34] Chuckran CA , Liu C , Bruno TC , Workman CJ , Vignali DA . Neuropilin‐1: a checkpoint target with unique implications for cancer immunology and immunotherapy. J Immunother Cancer. 2020;8(2):e000967. 32675311 10.1136/jitc-2020-000967 PMC7368550

[35] Xin Y , Li J , Wu J , et al. Pharmacokinetic and pharmacodynamic analysis of circulating biomarkers of anti‐NRP1, a novel antiangiogenesis agent, in two phase I trials in patients with advanced solid tumors. Clin Cancer Res. 2012;18(21):6040‐6048. 22962439 10.1158/1078-0432.CCR-12-1652

[36] Ku HC , Cheng CF . Master regulator activating transcription factor 3 (ATF3) in metabolic homeostasis and cancer. Front Endocrinol (Lausanne). 2020;11:556. 32922364 10.3389/fendo.2020.00556 PMC7457002

[37] Asakawa Y , Okabe A , Fukuyo M , et al. Epstein‐Barr virus‐positive gastric cancer involves enhancer activation through activating transcription factor 3. Cancer Sci. 2020;111(5):1818‐1828. 32119176 10.1111/cas.14370 PMC7226279

[38] Huang C , Chen R , Zheng F , et al. Inhibitory role of ATF3 in gastric cancer progression through regulating cell EMT and stemness. Cancer Cell Int. 2021;21(1):127. 33608016 10.1186/s12935-021-01828-9 PMC7893881

[39] Fei P , Wang W , Kim SH , et al. Bnip3L is induced by p53 under hypoxia, and its knockdown promotes tumor growth. Cancer Cell. 2004;6(6):597‐609. 15607964 10.1016/j.ccr.2004.10.012

[40] Arao T , Yanagihara K , Takigahira M , et al. ZD6474 inhibits tumor growth and intraperitoneal dissemination in a highly metastatic orthotopic gastric cancer model. Int J Cancer. 2006;118(2):483‐489. 16052530 10.1002/ijc.21340

[41] Zhao X , Cai H , Wang X , Ma L . Discovery of signature genes in gastric cancer associated with prognosis. Neoplasma. 2016;63(2):239‐245. 26774142 10.4149/209_150531N303

[42] Thomassen M , Tan Q , Kruse TA . Gene expression meta‐analysis identifies metastatic pathways and transcription factors in breast cancer. BMC Cancer. 2008;8:394. 19116006 10.1186/1471-2407-8-394 PMC2642844

[43] Xiao L , Zhou J , Liu H , et al. RNA sequence profiling reveals unique immune and metabolic features of breast cancer brain metastases. Front Oncol. 2021;11:679262. 34513670 10.3389/fonc.2021.679262 PMC8427193

[44] Yang J , Lin P , Yang M , et al. Integrated genomic and transcriptomic analysis reveals unique characteristics of hepatic metastases and pro‐metastatic role of complement C1q in pancreatic ductal adenocarcinoma. Genome Biol. 2021;22(1):4. 33397441 10.1186/s13059-020-02222-w PMC7780398

[45] Saitoh M . Epithelial‐mesenchymal transition is regulated at post‐transcriptional levels by transforming growth factor‐β signaling during tumor progression. Cancer Sci. 2015;106(5):481‐488. 25664423 10.1111/cas.12630 PMC4452147

[46] Chen Y , Gong W , Dai W , Jiang H , Xu X . E2F1/2/4 mRNA is associated with immune infiltration and are potential biomarkers for the prognosis of human gastric carcinoma. Transl Cancer Res. 2021;10(6):2801‐2811. 35116590 10.21037/tcr-21-45 PMC8797903

[47] Yan L‐H , Chen Z‐N , Li L , et al. E2F‐1 promotes DAPK2‐induced anti‐tumor immunity of gastric cancer cells by targeting miR‐34a. Tumor Biol. 2016;37(12):15925‐15936. 10.1007/s13277-016-5446-7 27704360

[48] Yang Y , Zhang J , Chen Y , Xu R , Zhao Q , Guo W . MUC4, MUC16, and TTN genes mutation correlated with prognosis, and predicted tumor mutation burden and immunotherapy efficacy in gastric cancer and pan‐cancer. Clin Transl Med. 2020;10(4):e155. 32898332 10.1002/ctm2.155 PMC7443139

[49] Bi F , Chen Y , Yang Q . Significance of tumor mutation burden combined with immune infiltrates in the progression and prognosis of ovarian cancer. Cancer Cell Int. 2020;20:373. 32774167 10.1186/s12935-020-01472-9 PMC7405355

[50] Havel JJ , Chowell D , Chan TA . The evolving landscape of biomarkers for checkpoint inhibitor immunotherapy. Nat Rev Cancer. 2019;19(3):133‐150. 30755690 10.1038/s41568-019-0116-x PMC6705396

[51] Le DT , Uram JN , Wang H , et al. PD‐1 blockade in tumors with mismatch‐repair deficiency. N Engl J Med. 2015;372(26):2509‐2520. 26028255 10.1056/NEJMoa1500596 PMC4481136

[52] Tian Y , Xu J , Chu Q , et al. A novel tumor mutational burden estimation model as a predictive and prognostic biomarker in NSCLC patients. BMC Med. 2020;18(1):232. 32843031 10.1186/s12916-020-01694-8 PMC7448445

[53] Guo X , Liang X , Wang Y , et al. Significance of tumor mutation burden combined with immune infiltrates in the progression and prognosis of advanced gastric cancer. Front Genet. 2021;12:642608. 34306002 10.3389/fgene.2021.642608 PMC8299211

[54] Simon S , Labarriere N . PD‐1 expression on tumor‐specific T cells: friend or foe for immunotherapy? Onco Targets Ther. 2017;7(1):e1364828. 10.1080/2162402X.2017.1364828 PMC5739549 29296515

[55] Wang YL , Gong Y , Lv Z , Li L , Yuan Y . Expression of PD1/PDL1 in gastric cancer at different microsatellite status and its correlation with infiltrating immune cells in the tumor microenvironment. J Cancer. 2021;12(6):1698‐1707. 33613757 10.7150/jca.40500 PMC7890312

[56] Liu J , Li H , Sun L , Yuan Y , Xing C . Profiles of PD‐1, PD‐L1, PD‐L2 in gastric cancer and their relation with mutation, immune infiltration, and survival. Biomed Res Int. 2020;2020:2496582. 32596285 10.1155/2020/2496582 PMC7298268

[57] Patel SP , Kurzrock R . PD‐L1 expression as a predictive biomarker in cancer immunotherapy. Mol Cancer Ther. 2015;14(4):847‐856. 25695955 10.1158/1535-7163.MCT-14-0983

[58] Conroy JM , Pabla S , Nesline MK , et al. Next generation sequencing of PD‐L1 for predicting response to immune checkpoint inhibitors. J Immunother Cancer. 2019;7(1):18. 30678715 10.1186/s40425-018-0489-5 PMC6346512

[59] Jiang P , Gu S , Pan D , et al. Signatures of T cell dysfunction and exclusion predict cancer immunotherapy response. Nat Med. 2018;24(10):1550‐1558. 30127393 10.1038/s41591-018-0136-1 PMC6487502

[60] Wang S , He Z , Wang X , Li H , Liu XS . Antigen presentation and tumor immunogenicity in cancer immunotherapy response prediction. Elife. 2019;8:e49020. 31767055 10.7554/eLife.49020 PMC6879305

[61] Kim J , Kim B , Kang SY , et al. Tumor mutational burden determined by panel sequencing predicts survival after immunotherapy in patients with advanced gastric cancer. Front Oncol. 2020;10:314. 32232003 10.3389/fonc.2020.00314 PMC7082319

[62] Ratti M , Lampis A , Hahne JC , Passalacqua R , Valeri N . Microsatellite instability in gastric cancer: molecular bases, clinical perspectives, and new treatment approaches. Cell Mol Life Sci. 2018;75(22):4151‐4162. 30173350 10.1007/s00018-018-2906-9 PMC6182336

[63] Chen B , Khodadoust MS , Liu CL , Newman AM , Alizadeh AA . Profiling tumor infiltrating immune cells with CIBERSORT. Methods Mol Biol. 2018;1711:243‐259. 29344893 10.1007/978-1-4939-7493-1_12 PMC5895181

[64] Baumjohann D , Brossart P . T follicular helper cells: linking cancer immunotherapy and immune‐related adverse events. J Immunother Cancer. 2021;9(6):e002588. 34112740 10.1136/jitc-2021-002588 PMC8194326

[65] Ahearne MJ , Allchin RL , Fox CP , Wagner SD . Follicular helper T‐cells: expanding roles in T‐cell lymphoma and targets for treatment. Br J Haematol. 2014;166(3):326‐335. 24815671 10.1111/bjh.12941

[66] Sousa S , Brion R , Lintunen M , et al. Human breast cancer cells educate macrophages toward the M2 activation status. Breast Cancer Res. 2015;17(1):101. 26243145 10.1186/s13058-015-0621-0 PMC4531540

[67] Hwang I , Kim JW , Ylaya K , et al. Tumor‐associated macrophage, angiogenesis and lymphangiogenesis markers predict prognosis of non‐small cell lung cancer patients. J Transl Med. 2020;18(1):443. 33228719 10.1186/s12967-020-02618-z PMC7686699

[68] Cheng H , Wang Z , Fu L , Xu T . Macrophage polarization in the development and progression of ovarian cancers: an overview. Front Oncol. 2019;9:421. 31192126 10.3389/fonc.2019.00421 PMC6540821

[69] Gambardella V , Castillo J , Tarazona N , et al. The role of tumor‐associated macrophages in gastric cancer development and their potential as a therapeutic target. Cancer Treat Rev. 2020;86:102015. 32248000 10.1016/j.ctrv.2020.102015

