# Supplementary figures and images for: Construction of ovarian metastasis‐related immune signature predicting prognosis of gastric cancer patients
Source: Cancer Med. 2022 May 27;12(1):913–29. doi: 10.1002/cam4.4857 (PMC9844635; doi:10.1002/cam4.4857)

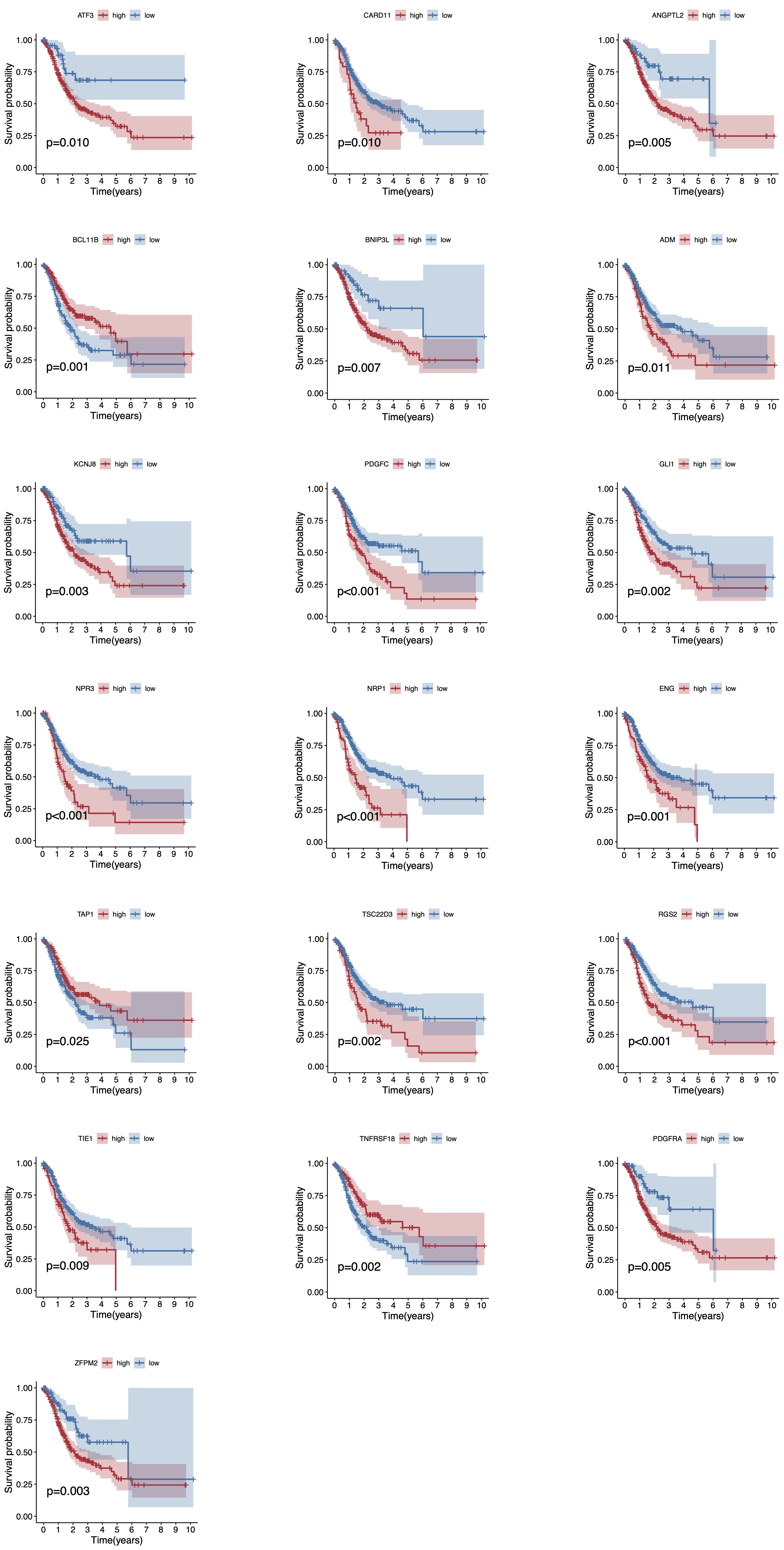

Supplement: Supplementary file 1 — Figure S1 [file CAM4-12-913-s006.jpg]

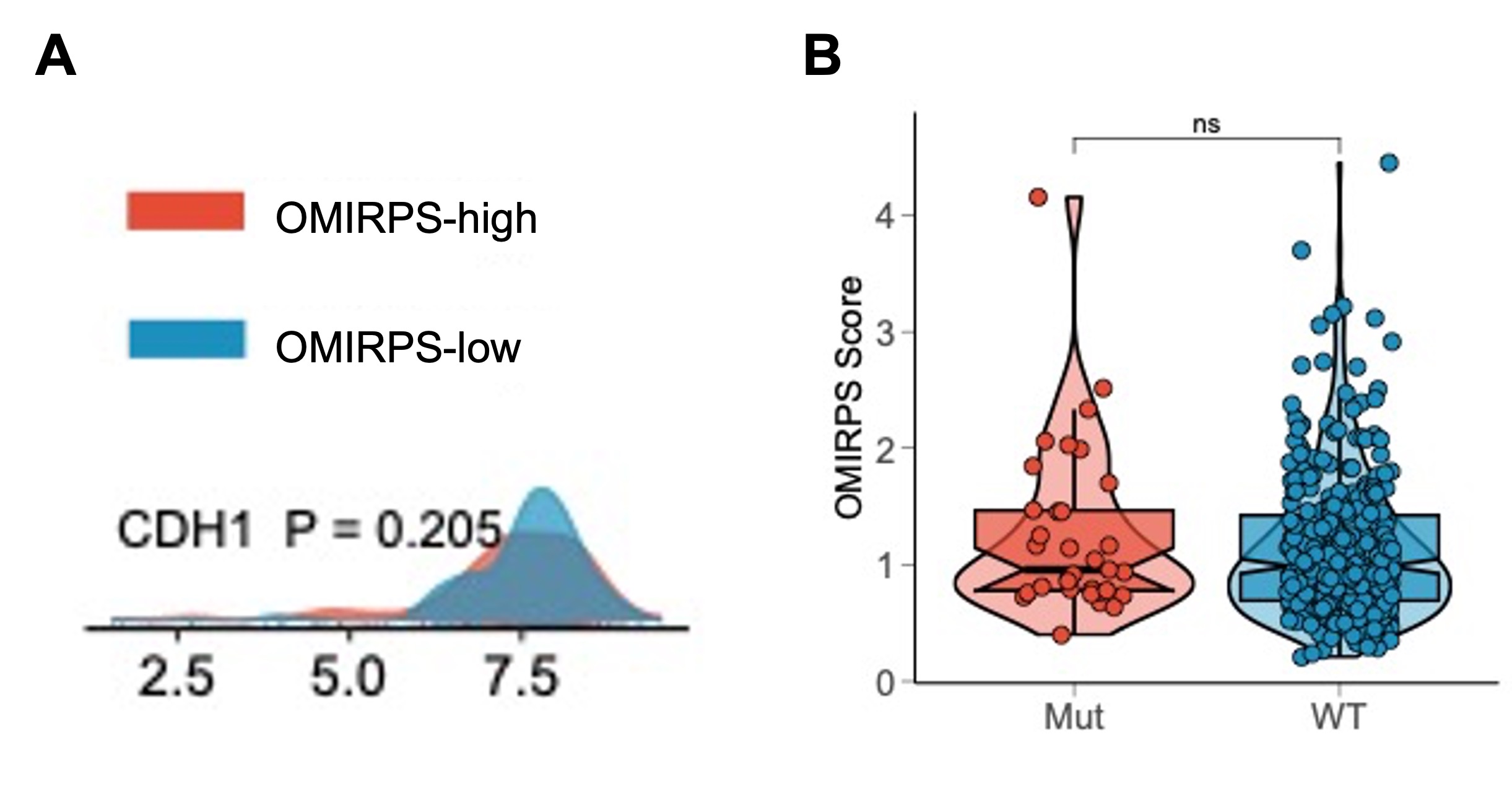

Supplement: Supplementary file 2 — Figure S2 [file CAM4-12-913-s002.jpg]

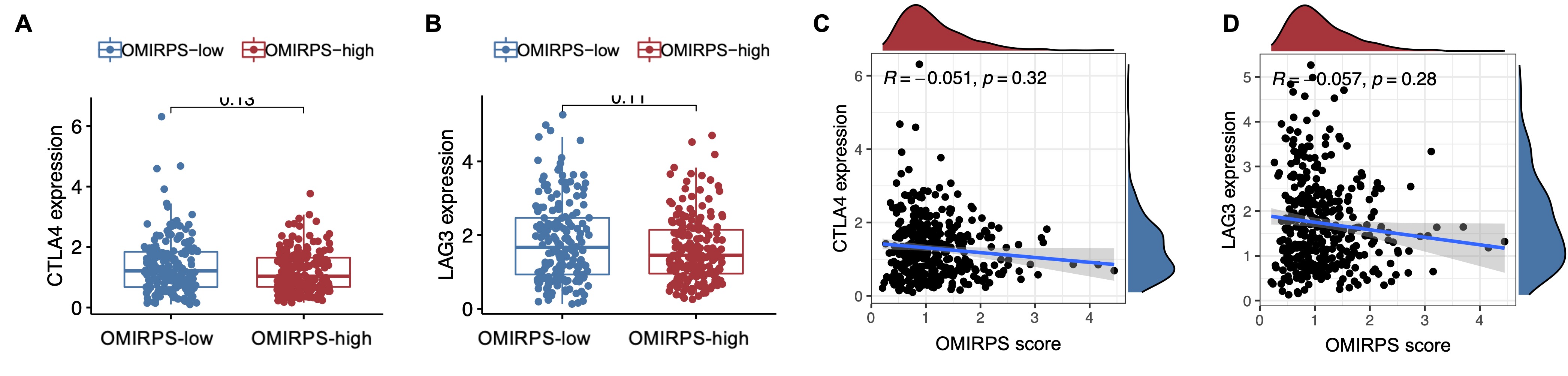

Supplement: Supplementary file 3 — Figure S3 [file CAM4-12-913-s004.jpg]

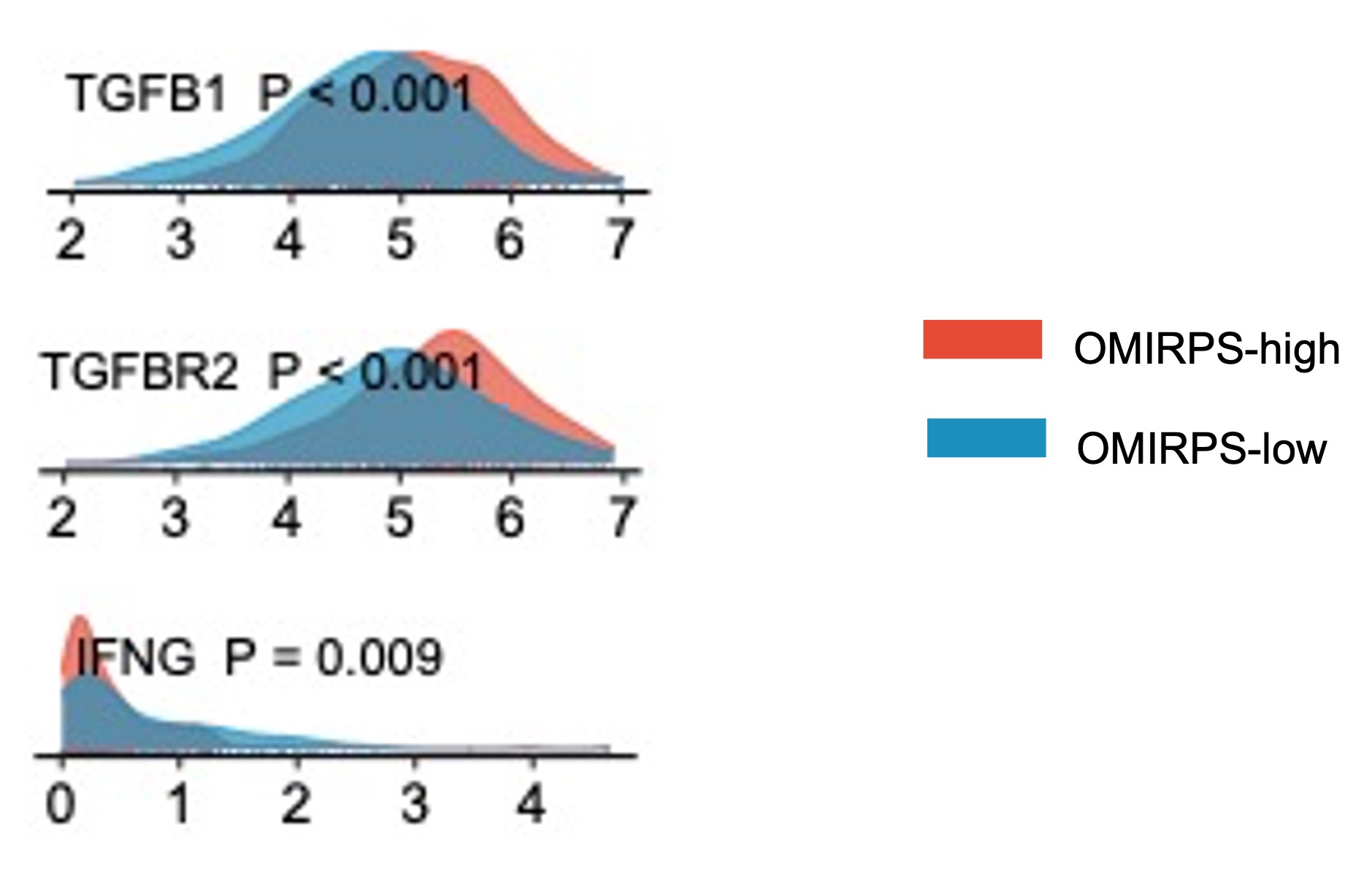

Supplement: Supplementary file 4 — Figure S4 [file CAM4-12-913-s003.jpg]

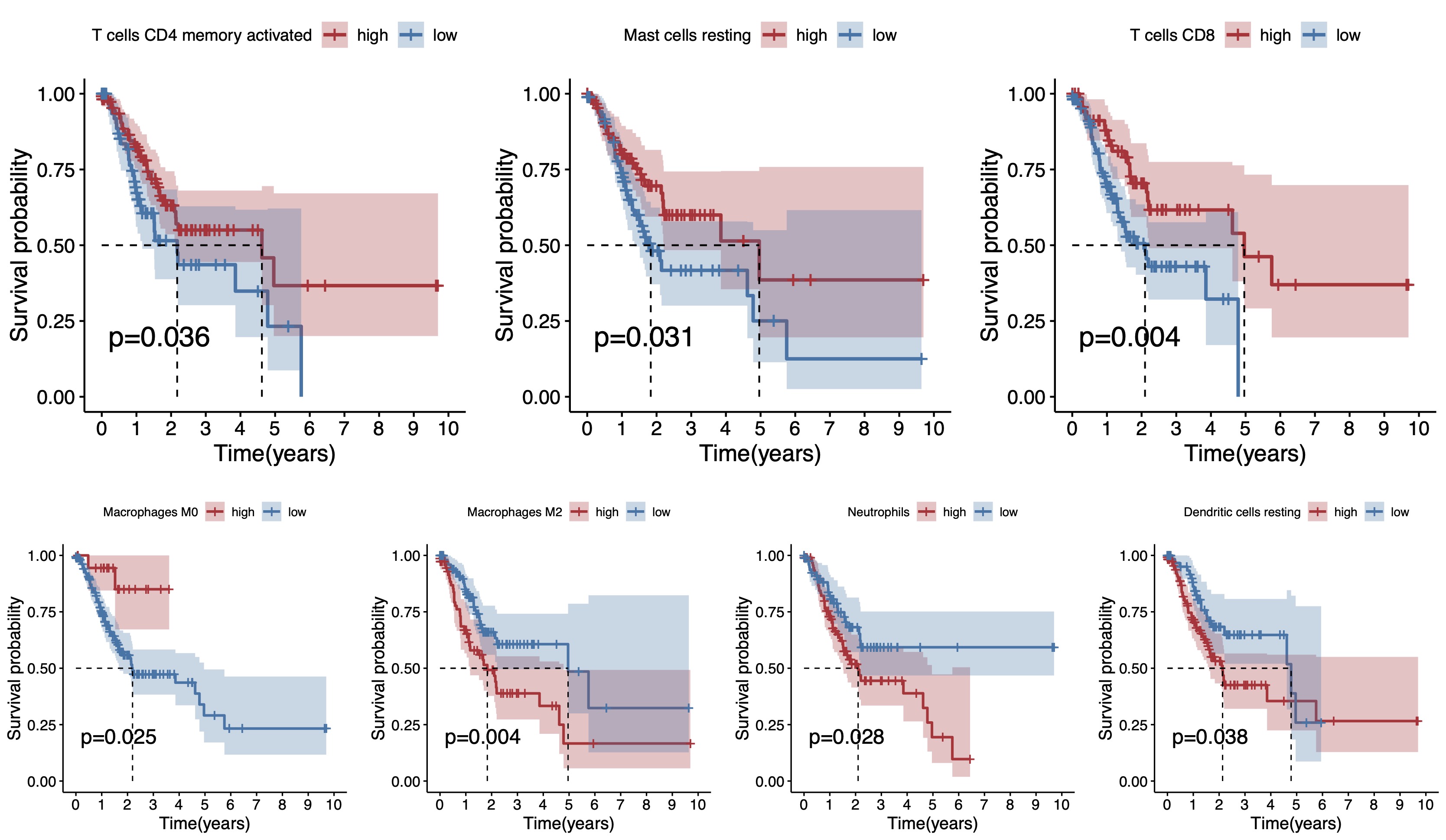

Supplement: Supplementary file 5 — Figure S5 [file CAM4-12-913-s005.jpg]
