# Supplementary material for: Construction of ovarian metastasis‐related immune signature predicting prognosis of gastric cancer patients
Source: Cancer Med. 2022 May 27;12(1):913–29. doi: 10.1002/cam4.4857 (PMC9844635; doi:10.1002/cam4.4857)
Supplement: Supplementary file 6 — Table S1–S3 [file CAM4-12-913-s001.docx]

**Table S1. Top Enriched GO Categories of Significantly Dysregulated Genes Between Primary GC and OM**

| **GO Term in Biological Process** | **Description** | **BgRatio** | **p.adjust** | **Q value** | **Number of genes** |
| --- | --- | --- | --- | --- | --- |
| GO:0042110 | T cell activation | 483/18866 | 5.7013E-19 | 4.61428E-19 | 102 |
| GO:0030098 | Lymphocyte differentiation | 368/18866 | 7.92733E-15 | 6.41589E-15 | 79 |
| GO:0030198 | Extracellular matrix organization | 395/18866 | 1.11814E-12 | 9.04955E-13 | 78 |
| GO:0022407 | Regulation of cell-cell adhesion | 439/18866 | 1.60605E-10 | 1.29984E-10 | 78 |
| GO:0022604 | Regulation of cell morphogenesis | 499/18866 | 8.91979E-06 | 7.21913E-06 | 71 |
| GO:0007159 | Leukocyte cell-cell adhesion | 364/18866 | 6.90078E-11 | 5.58506E-11 | 70 |
| GO:0034329 | Cell junction assembly | 434/18866 | 2.60078E-06 | 2.10491E-06 | 66 |
| GO:0016049 | Cell growth | 490/18866 | 9.13348E-05 | 7.39207E-05 | 66 |
| GO:0002429 | Immune response-activating cell surface receptor signaling pathway | 481/18866 | 9.45862E-05 | 7.65522E-05 | 65 |
| GO:0043087 | Regulation of GTPase activity | 481/18866 | 0.000177704 | 0.000143823 | 64 |

**Table S2. Immune-related genes (IRGs) from the ImmPort and InnateDB databases**

| **ImmPort** | **InnateDB** |
| --- | --- |
| CD1D | Nod2 |
| CD8A | EFTUD2 |
| CREB1 | Tlr9 |
| CTSB | Ifnb1 |
| CTSS | IRF3 |
| HLA-E | CASP1 |
| HSPA1A | MIR223 |
| HSPA1B | COLEC12 |
| HSP90AA1 | Ccl5 |
| ICAM1 | IFNG |
| IFNA1 | DDX60L |
| IFNA2 | HDAC2 |
| IFNG | RORC |
| KIR3DL2 | Map3k8 |
| LGMN | SKP2 |
| PSMB8 | IFI27 |
| RELB | Trpm5 |
| THBS1 | Sc4mol |
| AP3B1 | Mir342 |
| PROCR | Cd48 |
| CD209 | Il18 |
| ERAP1 | Atg5 |
| HAMP | Zbp1 |
| CAMP | Zfp36 |
| REG3G | Nfkb2 |
| CXCL14 | Ybx1 |
| CXCL16 | SYK |
| CXCL10 | Ager |
| CXCL9 | S100a9 |
| CXCL5 | S100a8 |
| CXCL11 | Lcp2 |
| CXCL1 | Tirap |
| CXCL12 | Il17rd |
| CXCL13 | DHX33 |
| CXCL2 | RNASEL |
| DEFB103B | MIR362 |
| CCL1 | Mir125a |
| DEFB1 | Ptpn6 |
| ELANE | CTSS |
| DEFB103A | Mir328 |
| DEFA3 | Il4 |
| DEFA1 | Nlrp12 |
| DEFA6 | DEFB103A |
| DEFA5 | DEFB4B |
| DEFA4 | DEFB4 |
| LCN2 | Mapk14 |
| S100A9 | FCN3 |
| S100A8 | Icosl |
| DCD | Smpdl3b |
| S100A12 | Gsk3a |
| MMP12 | Pycard |
| SFTPD | MAP3K7 |
| PGLYRP1 | PDE12 |
| ZC3HAV1 | PQBP1 |
| PGLYRP3 | Tollip |
| PGLYRP2 | Pik3c3 |
| S100A10 | Cav1 |
| PGLYRP4 | Myo18a |
| COLEC12 | Il1rl1 |
| MAVS | IL1RAPL1 |
| DEFB4B | Irf5 |
| IFNAR1 | TPP2 |
| IFNGR1 | Ptpn11 |
| IL6 | Icam1 |
| TGFB1 | TP53 |
| MMP9 | Arl5b |
| TLR4 | IL22RA2 |
| NFKB1 | TLR6 |
| APOBEC3G | Cxcr6 |
| NOD2 | DEFA6 |
| MBL2 | DEFA5 |
| SFTPA1 | Il1r2 |
| TLR2 | Adam17 |
| IL1B | Il28ra |
| PLTP | MIR181A2 |
| MX1 | Tyrobp |
| DDX58 | Relb |
| IRF3 | Ripk3 |
| SFTPA2 | ATF2 |
| NOX4 | Slc11a1 |
| IFNB1 | Prl |
| DUOX1 | Adrbk1 |
| C8G | MFF |
| SPAG11A | DNM1L |
| NOX1 | Mir302b |
| TNF | Kdm4a |
| CTSG | Vegfc |
| PRTN3 | Flt4 |
| MAPK1 | Nlrp1a |
| PML | Hdac1 |
| CYBB | Il12b |
| ISG20 | MYH9 |
| BCL3 | HNRNPL |
| DUOX2 | Mir149 |
| TLR3 | Sykb |
| IFIH1 | Krt16 |
| TRIM5 | MIR208B |
| IDO1 | MIR499A |
| ADIPOQ | AICDA |
| STAT3 | PARD3 |
| STAT1 | CD1D |
| SOCS3 | MSR1 |
| TNFSF10 | BGN |
| SOCS1 | CMA1 |
| RNASEL | C3 |
| IRF1 | C3ar1 |
| IL15 | Rheb |
| CD40 | Mir126 |
| TLR7 | Stat4 |
| PPIA | ARF6 |
| NLRX1 | CTSL1 |
| VEGFA | ELF4 |
| IKBKE | Dhx58 |
| ISG15 | JAK1 |
| DHX58 | TNK1 |
| TNFAIP3 | Reg3g |
| FCN2 | SLX4 |
| F2R | Bcl2 |
| IL27 | Il27 |
| CCL5 | HIF1AN |
| LEP | Ifi202b |
| CYLD | Il9 |
| MAPK14 | XRCC6 |
| JUN | PRKDC |
| ITGAV | Mfn2 |
| IRF5 | F2RL1 |
| CCR6 | PRTN3 |
| IL12B | Marco |
| TLR8 | CEBPB |
| CD81 | Nos2 |
| EIF2AK2 | IL28A |
| NOD1 | TRIM25 |
| MAPK8 | RNF135 |
| MAPK3 | IKBKG |
| BST2 | Traf5 |
| PLA2G2A | Axl |
| GRN | Tyro3 |
| ADAR | CXCL12 |
| TYK2 | MIR141 |
| NOS2 | PIAS3 |
| TRAF3 | MIR21 |
| TLR1 | Zbtb20 |
| MX2 | MASP1 |
| F2RL1 | Sarm1 |
| MSR1 | UCHL1 |
| NFKBIZ | Klf4 |
| SRC | SRC |
| ELAVL1 | MIR15B |
| SOD1 | Ppp1cc |
| SLC11A1 | MIR517A |
| DMBT1 | Ifne |
| DAXX | Plunc |
| SPINK5 | TRIM42 |
| MARCO | TRIM38 |
| BECN1 | TRIM37 |
| TNFSF11 | TRIM36 |
| CSK | TRIM24 |
| JAK1 | TRIM67 |
| IRF7 | TRIM13 |
| IREB2 | TRIM9 |
| IL18 | TRIM71 |
| IL17A | TRIM58 |
| LTB4R | TRIM47 |
| APOBEC3A | TRIM32 |
| MASP2 | TRIM26 |
| TRIM27 | TRIM21 |
| RELA | MID1 |
| IL7R | TRIM15 |
| IL1A | TRIM7 |
| PTX3 | MUL1 |
| PTGS2 | Scaf11 |
| CD14 | Lum |
| MASP1 | Tacr1 |
| MAP2K2 | Siglecg |
| MAP2K1 | CNOT8 |
| HRG | Chat |
| IRF9 | Abl1 |
| TRIM22 | Prkcd |
| PPP4C | Mir212 |
| HMOX1 | Mir132 |
| HMGB1 | hsa-mir-132 |
| RNASE7 | Cryab |
| HDAC1 | ITGB3 |
| PLSCR1 | Fcnb |
| TANK | Glrx |
| PIK3CG | Hspa1b |
| RSAD2 | MOV10 |
| TBK1 | MIR187 |
| PDCD1 | AI607873 |
| APOH | CALCOCO2 |
| BIRC5 | MAP1LC3C |
| IL22 | VTRNA2-1 |
| GBP2 | TP73 |
| OAS1 | Tlr13 |
| AGER | Nlrc3 |
| UNC93B1 | MIR378 |
| TNFSF4 | Cftr |
| ACO1 | Itgb2 |
| CXCR1 | Ms4a8a |
| CCL17 | Pura |
| CCR3 | Edil3 |
| CCR7 | P2ry14 |
| CCL2 | Angpt1 |
| CXCR4 | Ly96 |
| CXCR6 | LILRA2 |
| CCR4 | Clec9a |
| PTK2B | Sftpa1 |
| IL4 | 1700021K19Rik |
| IL13 | TMED7;TICAM2 |
| IL10 | Il1rap |
| IL2 | Sdc4 |
| PPARG | Cd209a |
| MIF | Ntn1 |
| CRP | Nampt |
| JAK2 | Jak3 |
| PTK2 | Sharpin |
| CD86 | WNT3A |
| VDR | MIR16-2 |
| LYN | Trib2 |
| SYK | Apoa1 |
| BTK | Jak2 |
| RAC1 | Lrrk2 |
| RAC2 | Hp |
| PPP3CA | Plcg2 |
| PPP3R1 | Igf1 |
| NFAT5 | Gas6 |
| NFATC2 | Cd300a |
| NFATC3 | Rictor |
| NFATC4 | Ccl17 |
| HRAS | Ifit2 |
| NRAS | mmu-mir-10a |
| BCL10 | Rgs2 |
| MALT1 | Nox1 |
| CHUK | Ppp3r1 |
| IKBKB | Pcbp1 |
| IKBKG | Trp53 |
| NFKBIA | Fancc |
| NFKBIB | APOBEC3B |
| NFKBIE | Pglyrp3 |
| CR2 | Dcn |
| PIK3R1 | MIR125B1 |
| PIK3CA | Rac2 |
| PIK3CB | Pklr |
| PIK3CD | Senp2 |
| AKT1 | Pla2g4a |
| GSK3B | Gzmm |
| INPP5D | SELK |
| CD22 | Aire |
| PTPN6 | Ppargc1b |
| LILRB3 | Mapkapk2 |
| PLCG2 | Gata6 |
| IFITM1 | Tyk2 |
| C3 | TRAIP |
| C5 | TRADD |
| CMA1 | PTK2 |
| EDN1 | ZMYND11 |
| SEMA3A | CCDC88A |
| C5AR1 | MAFB |
| CXCR3 | CTNNAL1 |
| FPR1 | TNFRSF1B |
| FPR2 | GRK5 |
| GPR33 | FBXW5 |
| CXCR2 | RUNX3 |
| PLAUR | MAP3K7IP2 |
| PLXNA4 | GNAI3 |
| PTAFR | hsa-mir-146b |
| AREG | Itpr3 |
| AVP | Chga |
| CALCA | Oas1h |
| CHGA | BCL10 |
| CSF2 | LAT |
| FGF7 | HMGB2 |
| IFNE | KIR3DL2 |
| IGF1 | LGALS8 |
| IL12A | HERC5 |
| IL17C | NOX4 |
| IL17F | CNPY3 |
| IL19 | BIRC5 |
| IL21 | GAB1 |
| IL23A | SLAMF1 |
| IL25 | YWHAE |
| IL31 | BTN3A1 |
| IL32 | IFI6 |
| IL33 | PTMA |
| IL5 | GNB2 |
| IL6ST | SPI1 |
| IL7 | NLRP7 |
| IL9 | GLI1 |
| KITLG | Padi4 |
| NAMPT | TNFRSF12A |
| OSM | BMX |
| PRL |  |
| RETNLB | Id2 |
| AIMP1 | Iltifb |
| SPP1 | PRKRA |
| TNFSF9 | DDX3X |
| TSLP | DDX58 |
| VEGFC | MIR136 |
| ADRB2 | Mir146 |
| ANGPT1 | Il1a |
| C3AR1 | IFIH1 |
| CSF1R | Tlr2 |
| CSF2RB | Ap3b1 |
| EGFR | Atf3 |
| EPOR | Nlrp3 |
| FLT4 | IL29 |
| IFNGR2 | TRAF6 |
| IGF1R | MAVS |
| IL13RA1 | IKBKB |
| IL2RB | RELA |
| IL17RD | Tbk1 |
| IL17RE | Ppp4c |
| IL18R1 | Myd88 |
| IL1R1 | Tlr7 |
| IL1R2 | Tslp |
| IL1RAP | Chuk |
| IL1RL1 | IFIT5 |
| IL1RL2 | Stat2 |
| IL20RA | Stat1 |
| IL20RB | Adar |
| IL22RA2 | Ticam1 |
| IL2RG | CAMP |
| IL4R | Casp2 |
| KDR | Pcbp2 |
| LGR4 | Ube2i |
| LTBR | Baiap2l1 |
| NR1H3 | IL1B |
| NR1H4 | TLR4 |
| NR3C1 | PELI3 |
| NR4A3 | Atf7 |
| RORA | TLR3 |
| RORC | CLEC4E |
| RXRA | Traf3 |
| S1PR1 | Usp25 |
| SDC4 | Irf1 |
| TACR1 | Cxcl10 |
| THRB | ITGB1 |
| TNFRSF12A | NFKBIZ |
| TNFRSF13B | IL6 |
| TNFRSF13C | TET2 |
| TNFRSF18 | March5 |
| TNFRSF1A | Bnip3l |
| TNFRSF1B | Bnip3 |
| TNFRSF9 | Isg15 |
| PTPN11 | Cdkn1a |
| ITGB2 | Nfia |
| TYROBP | JMJD6 |
| LCP2 | PRMT1 |
| LAT | IL23A |
| CD48 | IL2 |
| PRKCA | IL7R |
| SH2D1A | Lcn2 |
| BID | Ireb2 |
| PTPRC | Aco1 |
| CDC42 | TMEM173 |
| MAP3K8 | Nrip1 |
| MAP3K14 | Bcl11b |
| CTLA4 | Egfr |
| CBL | Areg |
| AZGP1 | Il33 |
| B2M | Card9 |
| CALR | Nlrc5 |
| CANX | C2 |
| CD1A | C4B |
| CD1B | Mir485 |
| CD1C | PLA2G2A |
| CD1E | IL22 |
| CD4 | Cd47 |
| CD8B | Clec4n |
| CD74 | Pik3cd |
| CTSE | IFIT3 |
| CTSL | MIR146A |
| FCER1G | HACE1 |
| FCGRT | Retnla |
| PDIA3 | Ifngr1 |
| HFE | Cxcl13 |
| HLA-A | Mrc1 |
| HLA-B | NFKBIA |
| HLA-C | TNFAIP3 |
| HLA-DMA | Il2rb |
| HLA-DMB | Ezh2 |
| HLA-DOA | Irf8 |
| HLA-DOB | Sppl3 |
| HLA-DPA1 | Ahr |
| HLA-DPB1 | Ido1 |
| HLA-DQA1 | Lgals3 |
| HLA-DQA2 | Il25 |
| HLA-DQB1 | Mb21d1 |
| HLA-DRA | TLR5 |
| HLA-DRB1 | CXCR1 |
| HLA-DRB3 | Stat6 |
| HLA-DRB4 | Dhcr7 |
| HLA-DRB5 | Idi1 |
| HLA-F | Srebf2 |
| HLA-G | Tgfb1 |
| HLA-H | Sod1 |
| MR1 | LGALS1 |
| HSPA1L | Aim2 |
| HSPA2 | Irf7 |
| HSPA4 | Cxcr3 |
| HSPA5 | Tnfsf4 |
| HSPA6 | Clec4d |
| HSPA8 | Mtap1s |
| HSP90AB1 | Nlrp6 |
| IFNA4 | IL10 |
| IFNA5 | STAT3 |
| IFNA6 | MIR29A |
| IFNA7 | Trim12c |
| IFNA8 | MX1 |
| IFNA10 | Lyn |
| IFNA13 | Dhx15 |
| IFNA14 | Il17a |
| IFNA16 | Ern1 |
| IFNA17 | C5 |
| IFNA21 | CEACAM8 |
| KIR2DL1 | C5AR1 |
| KIR2DL2 | Hs2st1 |
| KIR2DL3 | Dusp1 |
| KIR2DL4 | Cxcr4 |
| KIR2DS1 | Il7 |
| KIR2DS3 | AIP |
| KIR2DS4 | Ticam2 |
| KIR2DS5 | IL32 |
| KIR3DL1 | ELAVL1 |
| KLRC1 | CYLD |
| KLRC2 | Uvrag |
| KLRC3 | CTSK |
| KLRD1 | DEFB103B |
| LTA | Treml4 |
| CIITA | SPHK1 |
| MICA | Peli1 |
| MICB | Ctnnb1 |
| NFYA | Gsk3b |
| NFYB | TLR8 |
| NFYC | UBE2V2 |
| PSMC1 | UBE2N |
| PSMC2 | UBE2W |
| PSMC3 | TRIM5 |
| PSMC4 | OAS3 |
| PSMC5 | OAS1 |
| PSMC6 | Psen2 |
| PSMD1 | Stmn1 |
| PSMD2 | IRAK1 |
| PSMD3 | TIFA |
| PSMD4 | Rora |
| PSMD5 | IFRD1 |
| PSMD7 | TNF |
| PSMD8 | RNASE7 |
| PSMD10 | IFI16 |
| PSMD11 | MAPK8 |
| PSMD13 | Pten |
| PSME1 | Sqstm1 |
| PSME2 | TREM1 |
| RFX5 | PGLYRP1 |
| RFXAP | Il17f |
| SLC10A2 | Cxcl1 |
| TAP1 | Defb1 |
| TAP2 | Dnase2a |
| TAPBP | FPR2 |
| SEM1 | FPR1 |
| KLRC4 | PPARG |
| RFXANK | PPARGC1A |
| PSMD6 | MIR130A |
| PSME3 | Mir199a-1 |
| PSMD14 | Akt1 |
| CLEC4M | DR1 |
| IFI30 | CASP4 |
| ADRM1 | PTX3 |
| ECPAS | S100A12 |
| TRPC4AP | CXCL14 |
| UBXN1 | Rac1 |
| TAPBPL | Cd81 |
| KIR2DL5A | CAPRIN1 |
| ERAP2 | G3BP1 |
| ULBP3 | ULK1 |
| ULBP2 | Nfil3 |
| ULBP1 | Anpep |
| KIR3DL3 | WFDC12 |
| RAET1E | Ctnnd1 |
| RAET1L | Ly6g |
| UBR1 | HAVCR2 |
| RAET1G | HIF1A |
| PDIA2 | Mapk3 |
| PI3 | Mapk1 |
| DEFB4A | Csf1r |
| PPBP | Traf2 |
| SLPI | Cfp |
| CXCL8 | Hc |
| CXCL6 | Wdfy1 |
| PF4 | Ear11 |
| XCL1 | Tnfrsf13c |
| CXCL3 | Osm |
| CCL13 | Cxcl5 |
| CCL8 | Atm |
| TMSB10 | ZBTB16 |
| LCN1 | IL18R1 |
| COLEC10 | IL1F7 |
| BPI | Sf3a1 |
| LCN6 | CLEC6A |
| HTN3 | Cd200r1 |
| LCN8 | Cd200 |
| DEFA1B | ANO6 |
| CCR10 | P2RX7 |
| CELA1 | Zfp423 |
| DEFB106A | Rel |
| PENK | SLC22A3 |
| BPIFC | EIF2AK2 |
| BPIFB6 | RPS6KA5 |
| LEAP2 | IFITM3 |
| LCN9 | INSIG1 |
| BPIFB2 | AMFR |
| PTGDS | Eif4ebp2 |
| TMSB4X | Eif4ebp1 |
| TMSB15A | Anxa1 |
| S100B | Sirpa |
| S100A13 | Itgam |
| S100A6 | Clec7a |
| DEFB119 | Irak4 |
| DEFB107A | Cd40 |
| DEFB105A | Cd86 |
| SERPIND1 | Tmem126a |
| DEFB129 | Atg7 |
| DEFB127 | MIR548G |
| S100P | Cxcl2 |
| S100A7 | Cebpa |
| DEFB104A | Cxcl16 |
| DEFB126 | Il1r1 |
| DEFB106B | MIR23A |
| DEFB104B | Il15 |
| DEFB107B | Nlrc4 |
| S100A2 | Nod1 |
| DEFB125 | SMAD4 |
| DEFB123 | SMAD3 |
| DEFB105B | Nfkb1 |
| DEFB132 | ADCY8 |
| BPIFB3 | SCN5A |
| LCN12 | ECSIT |
| S100A11 | USP2 |
| S100A5 | Myc |
| S100A3 | ARG1 |
| S100A1 | Pik3cg |
| DEFB128 | Rab8a |
| DEFB108B | PRKX |
| HTN1 | PRKACA |
| LMBR1L | S1pr1 |
| S100A7A | CCNA2 |
| DEFB118 | MIR124-1 |
| TMSB4Y | PIK3CA |
| DEFB131A | MIR203 |
| DEFB134 | SAMHD1 |
| DEFB130A | Cxcr2 |
| DEFB124 | MIR122 |
| DEFB121 | hsa-mir-146a |
| DEFB116 | RIPK2 |
| DEFB115 | IRF4 |
| DEFB114 | Il20rb |
| DEFB113 | Il20ra |
| DEFB112 | Il19 |
| DEFB110 | Ptges |
| TMSB15B | BST2 |
| DEFB133 | MAPK9 |
| S100Z | MAP2K7 |
| TMSB4XP8 | Il5 |
| S100A14 | Il13 |
| LCN10 | Daxx |
| S100A16 | Prdm1 |
| DEFB136 | IFIT1 |
| DEFB135 | BTK |
| DEFB117 | TRIM14 |
| ZC3HAV1L | TXNIP |
| S100A7L2 | Cd5l |
| MBL3P | IL28B |
| BPIFB4 | Fscn1 |
| AZU1 | Cd36 |
| DEFB131B | HMGB1 |
| DEFA1A3 | HSPA1A |
| LCN1P1 | Tsc1 |
| S100G | Mtor |
| DEFA7P | Kdr |
| DEFB130B | Dusp16 |
| DEFB108F | GNB2L1 |
| DEFB131C | Nlrx1 |
| TCHHL1 | Bcl2l1 |
| TINAGL1 | Il17c |
| SLC22A17 | Gata3 |
| WFIKKN1 | EGLN2 |
| WFDC2 | CRKL |
| UMODL1 | Numb |
| PF4V1 | XRCC5 |
| ANOS1 | CREB1 |
| SPAG11B | Nfe2l2 |
| A2M | Keap1 |
| FABP6 | Mertk |
| RBP1 | LGALS9 |
| SLC40A1 | HDAC11 |
| PLAU | MIR145 |
| PAEP | PARK2 |
| HJV | POLR2F |
| MUC5AC | UCP2 |
| OBP2A | MIR133A1 |
| IFNL1 | IL12A |
| LPA | RNF125 |
| LBP | ASCC3 |
| RBP4 | MASP2 |
| LTF | WNT9B |
| RBP5 | WNT2B |
| FABP7 | VPS45 |
| FABP5 | ANKRD17 |
| FABP3 | Coch |
| FABP2 | Cdc42 |
| FABP4 | CD14 |
| R3HDML | Lgr4 |
| BPIFA3 | TNFSF10 |
| BPIFB1 | Ppp1ca |
| OASL | Psmb8 |
| CRABP2 | MIR517C |
| CRABP1 | Csf2 |
| RBP7 | Il21 |
| OBP2B | IKBKE |
| RBP2 | Mfge8 |
| LCN15 | MIR3148 |
| CETP | Serpinb2 |
| FABP12 | SFTPA1B;SFTPA1 |
| FABP9 | TRIM63 |
| BPIFA1 | TRIM61 |
| LCNL1 | TRIM60 |
| PI15 | TRIM55 |
| PMP2 | TRIM49 |
| APOD | TRIM45 |
| ORM2 | TRIM27 |
| ORM1 | TRIM23 |
| AEN | TRIM6 |
| BPIFA2 | MID2 |
| ISG20L2 | TRIM66 |
| NOX5 | TRIM65 |
| NOX3 | TRIM56 |
| TFRC | TRIM50 |
| LRP1 | TRIM8 |
| GDF15 | Gabarap |
| NEDD4 | MIRLET7B |
| IFNL2 | Mir135b |
| SEMG1 | Tlr1 |
| CCL20 | LEP |
| APOBEC3F | Naip5 |
| PLAAT4 | Fer |
| CHIT1 | Rad23a |
| ZYX | Nr1h4 |
| PGC | COX5B |
| TFR2 | Tbx21 |
| MUC4 | Drd2 |
| ELN | Tph1 |
| MAPT | ITGAV |
| LYZ | Fcna |
| KLKB1 | Mir497 |
| CST4 | Daglb |
| CSRP1 | TRIM62 |
| GNLY | Mir466l |
| APOM | Pydc3 |
| CACYBP | Pyhin1 |
| BPHL | BC094916 |
| NEWENTRY | Gm4955 |
| PDGFRA | TRIM28 |
| GNAI1 | Foxo3 |
| WNT5A | Adipoq |
| FURIN | Tnfaip8l2 |
| TPT1 | Abca1 |
| TPM2 | Mir467b |
| NEO1 | Trp73 |
| AHNAK | MIR10B |
| TK2 | Mcpt4 |
| PRDX2 | Mmp9 |
| FGF2 | Ahsg |
| FGA | Olfm4 |
| TCF7L2 | Itgax |
| TKFC | MS4A8B |
| LMBR1 | Casp7 |
| EPPIN | MIR1275 |
| MPO | MIR200C |
| ROBO3 | MIRLET7C |
| SP1 | Ace2 |
| PDF | Eif4e |
| DLL4 | NLRP4 |
| ECD | Zc3h12a |
| STING1 | Dusp10 |
| SKIV2L | E2f1 |
| SEMG2 | Rb1 |
| DES | Dok3 |
| DCK | Dicer1 |
| TNFRSF10A | Tufm |
| TNFRSF10B | mmu-mir-29a |
| EED | Gnai2 |
| CCL4 | Spink5 |
| LIMS1 | Lst1 |
| LALBA | Scarb1 |
| APOBEC3H | Spag11a |
| TMPRSS6 | Tsc22d3 |
| KNG1 | Hamp |
| KLRK1 | Rhbdf2 |
| KCNH2 | Plscr1 |
| JUND | Pacsin1 |
| CLDN4 | Irak3 |
| CCL28 | Eps8 |
| RNASE3 | Atf4 |
| RN7SL1 | Nfat5 |
| ILK | Ehmt2 |
| IFNAR2 | KIAA0226 |
| IFN1@ | Tmed7 |
| SYTL1 | CD209 |
| APOBEC3C | Camkk2 |
| DDX17 | Was |
| HTR1A | MIR16-1 |
| SEPTIN7 | Frem1 |
| CD40LG | Usp4 |
| PROC | DCD |
| NDRG1 | Fstl1 |
| LANCL1 | DEFA1 |
| ABCC4 | Hsp90b1 |
| HGF | Trpm2 |
| IFNLR1 | Itch |
| BACH2 | Il17re |
| ARRB1 | Unc5cl |
| STAB2 | Cnot4 |
| PDYN | Unc93b1 |
| PDGFRB | Serpinb9 |
| PCSK2 | Hsf1 |
| PCSK1 | Pros1 |
| ARG2 | Cd300lf |
| AQP9 | Yy1 |
| FASLG | MIR10A |
| ANXA6 | MRGPRX2 |
| VTN | Cdkn2a |
| VIM | MIR125B2 |
| VCAM1 | Dlk1 |
| PRDX1 | Tnip1 |
| GFAP | Fadd |
| ALB | Plaur |
| SLC29A3 | Apoh |
| NOS1 | Zfpm2 |
| ACTG1 | Zfpm1 |
| ACTA1 | Gata4 |
| SERPINA3 | Nr3c1 |
| CCL15 | Stub1 |
| CCL14 | Serpine1 |
| CCL16 | Tank |
| CCL19 | NAIP |
| CCL18 | Naip2 |
| CCL26 | Trem2 |
| CCL22 | APOBEC3G |
| CCL4L1 | Ddx41 |
| ACKR2 | Tax1bp1 |
| CCL27 | Neu1 |
| CCR8 | Aqp3 |
| ACKR4 | Elf1 |
| CCL21 | Cd8a |
| CCL7 | Muc1 |
| CCL3 | Il1rl2 |
| CCL11 | Rpl19 |
| CCR5 | Il4ra |
| CCL23 | IL4R |
| CCL25 | Sftpd |
| CCL3L3 | mmu-mir-29b-1 |
| CCL4L2 | Pltp |
| CCL3L1 | Ltbr |
| CCR1 | Rag1 |
| CCL24 | Tnfsf9 |
| XCL2 | Tnfrsf9 |
| TAFA5 | Snca |
| TAFA3 | F11 |
| TAFA4 | Tnfrsf1a |
| TAFA1 | Treml2 |
| TAFA2 | Cbl |
| CCL15-CCL14 | Hspd1 |
| CDH1 | Pml |
| LTBP1 | Pin1 |
| FGR | Hrg |
| PTGDR | Notch1 |
| HCK | Gbp2 |
| OLR1 | Hspa14 |
| GRK2 | Tgtp1 |
| TXK | Igtp |
| RNASE2 | Edn1 |
| CD79A | Socs1 |
| CD79B | Nras |
| BLNK | Irgm1 |
| VAV3 | Aimp1 |
| VAV1 | MIF |
| VAV2 | Gm16379 |
| RAC3 | Duox2 |
| PPP3CB | Rarres2 |
| PPP3CC | Kcnj8 |
| CHP1 | Nfatc4 |
| PPP3R2 | Nfatc3 |
| CHP2 | Clec1b |
| NFATC1 | Dhx36 |
| KRAS | Ddx21 |
| FOS | Ddx1 |
| CARD11 | Cd97 |
| CD19 | Adrb2 |
| PIK3R5 | Jam3 |
| PIK3R2 | Ubqln1 |
| PIK3R3 | Pmaip1 |
| AKT3 | Ip6k1 |
| AKT2 | Khsrp |
| CD72 | Ifnar1 |
| FCGR2B | Tpst1 |
| RASGRP3 | Plec |
| PRKCB | VENTX |
| IGH | Ccr3 |
| IGHA1 | Vldlr |
| IGHA2 | MIR23B |
| IGHD | Cflar |
| IGHD1-1 | Cdk6 |
| IGHD1-14 | MIR107 |
| IGHD1-20 | Gpr77 |
| IGHD1-26 | Lilrb3 |
| IGHD1-7 | Pik3ap1 |
| IGHD2-15 | Avp |
| IGHD2-2 | Casp8 |
| IGHD2-21 | SREBF1 |
| IGHD2-8 | Zc3hav1 |
| IGHD3-10 | Atg12 |
| IGHD3-16 | MIR373 |
| IGHD3-22 | MIR372 |
| IGHD3-3 | Akna |
| IGHD3-9 | IRAK2 |
| IGHD4-11 | CCL1 |
| IGHD4-17 | Pglyrp4 |
| IGHD4-23 | Gbp10 |
| IGHD4-4 | Gbp7 |
| IGHD5-12 | Gbp6 |
| IGHD5-18 | Gbp1 |
| IGHD5-24 | Tnfsf11 |
| IGHD5-5 | Arhgap15 |
| IGHD6-13 | Bid |
| IGHD6-19 | Thbs1 |
| IGHD6-25 | Tecpr1 |
| IGHD6-6 | HMGN2 |
| IGHD7-27 | Map3k5 |
| IGHE | Grn |
| IGHG1 | Cd46 |
| IGHG2 | FCN1 |
| IGHG3 | Nr1h3 |
| IGHG4 | Nr4a3 |
| IGHJ1 | H2-Ab1 |
| IGHJ2 | H2-Aa |
| IGHJ3 | Srxn1 |
| IGHJ4 | Gp2 |
| IGHJ5 | Hrh4 |
| IGHJ6 | Plg |
| IGHM | Ccl2 |
| IGHV1-18 | Impdh2 |
| IGHV1-2 | C1qc |
| IGHV1-24 | C1qb |
| IGHV1-3 | C1qa |
| IGHV1-45 | Ccbp2 |
| IGHV1-46 | Xiap |
| IGHV1-58 | Birc3 |
| IGHV1-69 | Birc2 |
| IGHV1-8 | Pglyrp2 |
| IGHV1-38-4 | Defb3 |
| IGHV1-69-2 | Raet1c |
| IGHV2-26 | Raet1a |
| IGHV2-5 | Crp |
| IGHV2-70 | Lgmn |
| IGHV3-11 | Cops5 |
| IGHV3-13 | Xbp1 |
| IGHV3-15 | Mbl2 |
| IGHV3-16 | Ccr6 |
| IGHV3-20 | Trp63 |
| IGHV3-21 | TP63 |
| IGHV3-23 | Cltc |
| IGHV3-30 | Fgf7 |
| IGHV3-30-3 | Cebpe |
| IGHV3-30-5 | MoleculeID 216094 |
| IGHV3-33 | CEBPD |
| IGHV3-35 | Coro2a |
| IGHV3-38 | Hmox1 |
| IGHV3-43 | TBKBP1 |
| IGHV3-48 | SIAH2 |
| IGHV3-49 | CD37 |
| IGHV3-53 | SLC15A4 |
| IGHV3-64 | SYP |
| IGHV3-66 | MAP2K6 |
| IGHV3-7 | MMP7 |
| IGHV3-72 | MMP12 |
| IGHV3-73 | NLRP2 |
| IGHV3-74 | NLRP9 |
| IGHV3-9 | NLRP11 |
| IGHV3-38-3 | NLRP13 |
| IGHV3-69-1 | NLRP8 |
| IGHV4-28 | NLRP5 |
| IGHV4-30-1 | PSMA7 |
| IGHV4-30-2 | TNFRSF18 |
| IGHV4-30-4 | CDK9 |
| IGHV4-31 | PTAFR |
| IGHV4-34 | C8A |
| IGHV4-39 | SELE |
| IGHV4-4 | C4A |
| IGHV4-59 | Mbl1 |
| IGHV4-61 | DEFA3 |
| IGHV4-38-2 | Apcs |
| IGHV5-51 | Cfh |
| IGHV5-10-1 | PTPN2 |
| IGHV6-1 | IFNGR2 |
| IGHV7-4-1 | RCAN1 |
| IGHV7-81 | SMAD7 |
| IGK | MALT1 |
| IGKC | ABCG1 |
| IGKDEL | SOCS6 |
| IGKJ | CSF2RB |
| IGKJ1 | LGALS2 |
| IGKJ2 | APOBEC3A |
| IGKJ3 | MAP3K7IP1 |
| IGKJ4 | MLST8 |
| IGKJ5 | TCEB2 |
| IGKV@ | MEFV |
| IGKV1-12 | PTK2B |
| IGKV1-13 | AMACR;C1QTNF3 |
| IGKV1-16 | SCARF1 |
| IGKV1-17 | CLEC4C |
| IGKV1-27 | SIGIRR |
| IGKV1-33 | HRAS |
| IGKV1-37 | C19orf29 |
| IGKV1-39 | C9 |
| IGKV1-5 | PIAS4 |
| IGKV1-6 | MAP2K2 |
| IGKV1-8 | SMAD6 |
| IGKV1-9 | STAP2 |
| IGKV1D-12 | ARRB2 |
| IGKV1D-13 | REST |
| IGKV1D-16 | HSP90AA1 |
| IGKV1D-17 | RCOR1 |
| IGKV1D-33 | KAT2B |
| IGKV1D-37 | NLRP1 |
| IGKV1D-39 | THRB |
| IGKV1D-42 | MTA1 |
| IGKV1D-43 | CCR4 |
| IGKV1D-8 | IL8 |
| IGKV2-24 | CD180 |
| IGKV2-28 | BCL2A1 |
| IGKV2-30 | TCEB1 |
| IGKV2-40 | VDR |
| IGKV2D-24 | SIAH1 |
| IGKV2D-28 | LPCAT2 |
| IGKV2D-29 | ACHE |
| IGKV2D-30 | MoleculeID 32847 |
| IGKV2D-40 | TNIP3 |
| IGKV3-11 | MAP3K12 |
| IGKV3-15 | ELMOD2 |
| IGKV3-20 | RNF41 |
| IGKV3-7 | BCAR1 |
| IGKV3D-11 | CD22 |
| IGKV3D-15 | CD274 |
| IGKV3D-20 | PDCD1LG2 |
| IGKV3D-7 | CCR7 |
| IGKV4-1 | SMARCE1 |
| IGKV5-2 | NKIRAS2 |
| IGKV6-21 | SOCS2 |
| IGKV6D-21 | BECN1 |
| IGKV6D-41 | NUMBL |
| IGL | RPS19 |
| IGLC1 | CAMK2A |
| IGLC2 | IFNA2 |
| IGLC3 | IFNA1 |
| IGLC6 | MAP3K14 |
| IGLC7 | ANXA4 |
| IGLJ | CYBB |
| IGLJ1 | BCL3 |
| IGLJ2 | RIPK1 |
| IGLJ3 | TRAFD1 |
| IGLJ4 | ITGA3 |
| IGLJ5 | IRF2BP1 |
| IGLJ6 | PIK3CB |
| IGLJ7 | LY86 |
| IGLV@ | RANBP9 |
| IGLV1-36 | NUP153 |
| IGLV1-40 | OTUD5 |
| IGLV1-44 | LILRA4 |
| IGLV1-47 | PROCR |
| IGLV1-50 | CASP12 |
| IGLV1-51 | CARD18 |
| IGLV10-54 | SOCS3 |
| IGLV11-55 | CYTIP |
| IGLV2-11 | Sirt1 |
| IGLV2-14 | FLI1 |
| IGLV2-18 | PTCH1 |
| IGLV2-23 | CTLA4 |
| IGLV2-33 | IL8RB |
| IGLV2-8 | AAMP |
| IGLV3-1 | NFATC2 |
| IGLV3-10 | COPS8 |
| IGLV3-12 | LRRFIP1 |
| IGLV3-16 | VEGFA |
| IGLV3-19 | NUP214 |
| IGLV3-21 | DMBT1 |
| IGLV3-22 | GPSM1 |
| IGLV3-25 | IRAK1BP1 |
| IGLV3-27 | KDM1 |
| IGLV3-32 | GJA1 |
| IGLV3-9 | MKNK1 |
| IGLV4-3 | MAP3K4 |
| IGLV4-60 | JUN |
| IGLV4-69 | CD53 |
| IGLV5-37 | SNX27 |
| IGLV5-39 | RUSC1 |
| IGLV5-45 | FCGR2A |
| IGLV5-48 | PTPRC |
| IGLV5-52 | MFN1 |
| IGLV6-57 | SPP1 |
| IGLV7-43 | C4bp |
| IGLV7-46 | C4BPB |
| IGLV8-61 | C4BPA |
| IGLV9-49 | Kitl |
| CCL3P1 | KITLG |
| CKLF | Pdcd1 |
| CX3CL1 | Rftn1 |
| CXCL17 | Hspbp1 |
| CCN1 | Akap10 |
| EDN2 | Ptges2 |
| EDN3 | Stim1 |
| FGF10 | Orai1 |
| LECT2 | Itpr1 |
| PPBPP1 | Lrrfip2 |
| PROK2 | Trib3 |
| SAA1 | Siglech |
| SAA2 | Siglece |
| SBDS | Siglec5 |
| SEMA3B | Siglec1 |
| SEMA3C | SIGLEC15 |
| SEMA3D | SIGLEC11 |
| SEMA3E | SIGLEC9 |
| SEMA3F | SIGLEC8 |
| SEMA3G | SIGLEC7 |
| SEMA4A | SIGLEC6 |
| SEMA4B | SIGLEC10 |
| SEMA4C | Rgmb |
| SEMA4D | 4432412L15Rik |
| SEMA4F | Oas2 |
| SEMA4G | Oas1g |
| SEMA5A | Oas1f |
| SEMA5B | Oas1e |
| SEMA6A | Oas1d |
| SEMA6B | Oas1c |
| SEMA6C | Oas1b |
| SEMA6D | Oas1a |
| SEMA7A | Ctsb |
| SLIT1 | Lair1 |
| SLIT2 | C8B |
| TNC | DEFA4 |
| TYMP | PIK3R1 |
| CCR9 | NLRP10 |
| CCRL2 | CARD16 |
| CMKLR1 | NFKBIE |
| CX3CR1 | OTUD7B |
| CXCR5 | CFB |
| ACKR3 | ISG20 |
| CYSLTR1 | IL2RG |
| CYSLTR2 | MST1R |
| ACKR1 | ADAM10 |
| EDNRA | MAP3K3 |
| EDNRB | NKIRAS1 |
| GPR17 | KLK1 |
| GPR32 | C1S |
| PTGDR2 | MAP3K1 |
| C5AR2 | PTGS2 |
| LTB4R2 | KPNA1 |
| PLXNA1 | C8G |
| PLXNA2 | C1R |
| PLXNA3 | LY9 |
| PLXNB1 | HLA-E |
| PLXNB2 | EGR1 |
| PLXNB3 | IL6ST |
| PLXNC1 | IGF1R |
| PLXND1 | ELP2 |
| ROBO1 | F2RL2 |
| ROBO2 | F2RL3 |
| RXFP3 | CD27 |
| XCR1 | HOXA9 |
| ADM | GSTP1 |
| ADM2 | EPOR |
| AGRP | WDR34 |
| AGT | ACAP1 |
| AMBN | ERAP1 |
| AMELX | CXCL11 |
| AMH | CXCL9 |
| ANGPTL5 | S100a10 |
| ANGPTL7 | Anxa2 |
| APLN | CEACAM1 |
| MANF | Tlr11 |
| CDNF | IL13RA1 |
| ARTN | Sema3a |
| BDNF | Plxna4 |
| BMP1 | CASP6 |
| BMP10 | Defa20 |
| BMP15 | PELI2 |
| BMP2 | hsa-mir-126 |
| BMP3 | FFAR2 |
| BMP4 | hsa-mir-98 |
| BMP5 | CISH |
| BMP6 | hsa-let-7e |
| BMP7 | HMGB3 |
| BMP8A | SIVA1 |
| BMP8B | SNAP23 |
| BTC | IRF2 |
| MYDGF | PLK1 |
| CALCB | TRAF1 |
| CAT | PKN1 |
| CCK | FXR1 |
| CD320 | ERBB2IP |
| CD70 | RBCK1 |
| ADA2 | RNF31 |
| CER1 | PIAS1 |
| CGA | WDR62 |
| CGB3 | DAB2IP |
| CGB1 | YJEFN3 |
| CGB2 | RP5-1000E10.4 |
| CGB5 | Trim30 |
| CGB7 | AZI2 |
| CGB8 | MAP3K7IP3 |
| CHGB | APOBEC3G;APOBEC3F |
| CLCF1 | hsa-mir-152 |
| CLEC11A | hsa-mir-148b |
| CMTM1 | hsa-mir-148a |
| CMTM2 | TRAT1 |
| CMTM3 | F2R |
| CMTM4 | FCGR1A |
| CMTM5 | UBD |
| CMTM6 | CD300E |
| CMTM7 | PRKCA |
| CMTM8 | CASP10 |
| CNTF | IRF6 |
| CORT | IRF9 |
| CRH | VASP |
| CSF1 | IFITM1 |
| CSF3 | IFITM2 |
| CSH1 | RAD21 |
| CSH2 | RASGEF1B |
| CSHL1 | NOXA1 |
| CSPG5 | TRPV2 |
| CTF1 | CYBA |
| CCN2 | CALCA |
| DKK1 | PPP3CA |
| EBI3 | OPTN |
| EGF | LGALS4 |
| EPGN | IL31 |
| EPO | RSAD2 |
| EREG | FCN2 |
| ESM1 | DUOX1 |
| FAM3B | Inpp5d |
| FAM3C | DHCR24 |
| FAM3D | Gpr33 |
| FGF1 | SFTPA2 |
| FGF11 | MIF;SLC2A11 |
| FGF12 | ILF3 |
| FGF13 | ATG9A |
| FGF14 | MX2 |
| FGF16 | TPSB2 |
| FGF17 | GOPC |
| FGF18 | LTB4R |
| FGF19 | BTN3A3 |
| FGF20 | BTN3A2 |
| FGF21 | Rxra |
| FGF22 | TOMM70A |
| FGF23 | USP17 |
| FGF3 | Ace |
| FGF4 | ETS1 |
| FGF5 | RAB11A |
| FGF6 | FZD1 |
| FGF8 | CTSG |
| FGF9 | ELANE |
| VEGFD | TNFRSF13B |
| FIGNL2 | DHX9 |
| FLT3LG | MAP2K1 |
| FSHB | DDIT3 |
| GAL | ATG16L1 |
| GALP | OTUB2 |
| GAST | OTUB1 |
| GCG | PPIA |
| GDF1 | XDH |
| GDF10 | CR2 |
| GDF11 | IFNAR2;IL10RB |
| GDF2 | Foxa2 |
| GDF3 | Defb14 |
| GDF5 | Fcrl5 |
| GDF6 | ING4 |
| GDF7 | Calm1 |
| GDF9 | NXN |
| GDNF | SPON2 |
| GH1 | CTCF |
| GH2 | RNF5 |
| GHRH | PIAS2 |
| GHRL | TCF4 |
| GIP | TLR10 |
| GKN1 | USP7 |
| GMFB | CARD6 |
| GMFG | C7 |
| GNRH1 | C6 |
| GNRH2 | BDKRB2 |
| GPHA2 | CTSD |
| GPHB5 | C1QBP |
| GPI | CSK |
| GREM1 | SCAMP5 |
| GREM2 | PSTPIP1 |
| GRP | CTSH |
| GUCA2A | TRIM22 |
| HBEGF | SMARCA4 |
| HDGF | NLRP14 |
| HDGFL3 | CCNT1 |
| IAPP | MAP2K4 |
| IFNK | MAP2K3 |
| IFNW1 | SUGT1 |
| IGF2 | NCKAP1L |
| IL11 | CD63 |
| IL16 | SMARCA2 |
| IL17B | SERPING1 |
| IL17D | RETNLB |
| IL1F10 | NFKBIB |
| IL36RN | PRKCE |
| IL36A | STAT5B |
| IL37 | STAT5A |
| IL36B | SOCS5 |
| IL36G | RPS6KA4 |
| IL1RN | SH2D1A |
| IL20 | SLAMF8 |
| IL24 | SLAMF9 |
| IL26 | SLAMF6 |
| IFNL3 | SLAMF7 |
| IL3 |  |
| IL34 |  |
| INHA |  |
| INHBA |  |
| INHBB |  |
| INHBC |  |
| INHBE |  |
| INS |  |
| INS-IGF2 |  |
| INSL3 |  |
| INSL4 |  |
| INSL5 |  |
| INSL6 |  |
| JAG1 |  |
| JAG2 |  |
| FGF7P6 |  |
| FGF7P3 |  |
| KL |  |
| LACRT |  |
| LEFTY1 |  |
| LEFTY2 |  |
| LHB |  |
| LIF |  |
| LRSAM1 |  |
| LTB |  |
| LTBP2 |  |
| LTBP3 |  |
| LTBP4 |  |
| MDK |  |
| MIA |  |
| MLN |  |
| MSTN |  |
| NDP |  |
| NENF |  |
| NGF |  |
| NMB |  |
| NODAL |  |
| CCN3 |  |
| NPFF |  |
| NPPA |  |
| NPPB |  |
| NPPC |  |
| NPY |  |
| NRG1 |  |
| NRG2 |  |
| NRG3 |  |
| NRG4 |  |
| NRTN |  |
| NTF3 |  |
| NTF4 |  |
| NTS |  |
| NUDT6 |  |
| OGN |  |
| OSGIN1 |  |
| OSTN |  |
| OXT |  |
| ENDOU |  |
| PDGFA |  |
| PDGFB |  |
| PDGFC |  |
| PDGFD |  |
| PDGFRL |  |
| PGF |  |
| PMCH |  |
| PNOC |  |
| POMC |  |
| PPBPP2 |  |
| PPY |  |
| PRLH |  |
| PROK1 |  |
| PSPN |  |
| PTH |  |
| PTH2 |  |
| PTHLH |  |
| PTN |  |
| PYY |  |
| QRFP |  |
| RABEP1 |  |
| RABEP2 |  |
| REG1A |  |
| RETN |  |
| RLN1 |  |
| RLN2 |  |
| RLN3 |  |
| SCG2 |  |
| SCGB3A1 |  |
| SCT |  |
| SECTM1 |  |
| SLURP1 |  |
| SST |  |
| STC1 |  |
| STC2 |  |
| TAC1 |  |
| TDGF1 |  |
| TDGF1P3 |  |
| TG |  |
| TGFA |  |
| TGFB2 |  |
| TGFB3 |  |
| THPO |  |
| TNFRSF11B |  |
| TNFSF12 |  |
| TNFSF13 |  |
| TNFSF13B |  |
| TNFSF14 |  |
| TNFSF15 |  |
| TNFSF18 |  |
| TNFSF8 |  |
| TOR2A |  |
| TRH |  |
| TSHB |  |
| TXLNA |  |
| UCN |  |
| UCN2 |  |
| UCN3 |  |
| UTS2 |  |
| UTS2B |  |
| VEGFB |  |
| VGF |  |
| VIP |  |
| ACVR1B |  |
| ACVR1C |  |
| ACVR2A |  |
| ACVR2B |  |
| ACVRL1 |  |
| ADCYAP1R1 |  |
| ADIPOR1 |  |
| ADIPOR2 |  |
| ADRB1 |  |
| AGTR1 |  |
| AGTR2 |  |
| AMHR2 |  |
| ANGPT4 |  |
| ANGPTL1 |  |
| ANGPTL2 |  |
| ANGPTL3 |  |
| ANGPTL4 |  |
| ANGPTL6 |  |
| APLNR |  |
| AR |  |
| AVPR1A |  |
| AVPR1B |  |
| AVPR2 |  |
| BMPR1A |  |
| BMPR1B |  |
| BMPR2 |  |
| BRD8 |  |
| CALCR |  |
| CALCRL |  |
| CNTFR |  |
| CRHR1 |  |
| CRHR2 |  |
| CRIM1 |  |
| CRLF1 |  |
| CRLF2 |  |
| CRLF3 |  |
| CSF2RA |  |
| CSF3R |  |
| ENG |  |
| ESR1 |  |
| ESR2 |  |
| ESRRA |  |
| ESRRB |  |
| ESRRG |  |
| FGFR1 |  |
| FGFR2 |  |
| FGFR3 |  |
| FGFR4 |  |
| FGFRL1 |  |
| FLT1 |  |
| FLT3 |  |
| FSHR |  |
| GALR2 |  |
| GALR3 |  |
| GCGR |  |
| GHR |  |
| GHRHR |  |
| GHSR |  |
| GIPR |  |
| GLP1R |  |
| GLP2R |  |
| GNRHR |  |
| GPER1 |  |
| HNF4A |  |
| HNF4G |  |
| HTR3A |  |
| HTR3B |  |
| HTR3C |  |
| HTR3D |  |
| HTR3E |  |
| IGF2R |  |
| IL10RA |  |
| IL10RB |  |
| IL11RA |  |
| IL12RB1 |  |
| IL12RB2 |  |
| IL13RA2 |  |
| IL15RA |  |
| IL17RA |  |
| IL17RB |  |
| IL17RC |  |
| IL18RAP |  |
| IL21R |  |
| IL22RA1 |  |
| IL23R |  |
| IL27RA |  |
| IL2RA |  |
| IL31RA |  |
| IL3RA |  |
| IL5RA |  |
| IL6R |  |
| IL9R |  |
| INSR |  |
| LEPR |  |
| LGR5 |  |
| LGR6 |  |
| LHCGR |  |
| LIFR |  |
| MC1R |  |
| MC2R |  |
| MC3R |  |
| MC4R |  |
| MCHR1 |  |
| MCHR2 |  |
| MET |  |
| MLNR |  |
| MPL |  |
| MTNR1A |  |
| MTNR1B |  |
| NGFR |  |
| NMBR |  |
| NPR1 |  |
| NPR3 |  |
| NR0B1 |  |
| NR0B2 |  |
| NR1D1 |  |
| NR1D2 |  |
| NR1H2 |  |
| NR1I2 |  |
| NR1I3 |  |
| NR2C1 |  |
| NR2C2 |  |
| NR2E1 |  |
| NR2E3 |  |
| NR2F1 |  |
| NR2F2 |  |
| NR2F6 |  |
| NR3C2 |  |
| NR4A1 |  |
| NR4A2 |  |
| NR5A1 |  |
| NR5A2 |  |
| NR6A1 |  |
| NRP1 |  |
| NRP2 |  |
| OGFR |  |
| OPRD1 |  |
| OPRK1 |  |
| OPRL1 |  |
| OPRM1 |  |
| OSMR |  |
| OXTR |  |
| PGR |  |
| PGRMC2 |  |
| PPARA |  |
| PPARD |  |
| PRLHR |  |
| PRLR |  |
| PTGER1 |  |
| PTGER2 |  |
| PTGER3 |  |
| PTGER4 |  |
| PTGFR |  |
| PTH1R |  |
| PTH2R |  |
| RARA |  |
| RARB |  |
| RARG |  |
| RORB |  |
| RXFP1 |  |
| RXFP2 |  |
| RXRB |  |
| RXRG |  |
| S1PR2 |  |
| SCTR |  |
| SDC1 |  |
| SDC2 |  |
| SDC3 |  |
| SORT1 |  |
| SSTR1 |  |
| SSTR2 |  |
| SSTR5 |  |
| ST2 |  |
| TEK |  |
| TGFBR1 |  |
| TGFBR2 |  |
| TGFBR3 |  |
| THRA |  |
| TIE1 |  |
| TNFRSF10C |  |
| TNFRSF10D |  |
| TNFRSF11A |  |
| TNFRSF14 |  |
| TNFRSF17 |  |
| TNFRSF19 |  |
| TNFRSF21 |  |
| TNFRSF25 |  |
| TNFRSF4 |  |
| TNFRSF6B |  |
| TNFRSF8 |  |
| TRHR |  |
| TSHR |  |
| TUBB3 |  |
| VIPR1 |  |
| VIPR2 |  |
| ICAM2 |  |
| ITGAL |  |
| PAK1 |  |
| NCR2 |  |
| LCK |  |
| FCGR3A |  |
| FCGR3B |  |
| NCR1 |  |
| NCR3 |  |
| CD247 |  |
| ZAP70 |  |
| PLCG1 |  |
| SH3BP2 |  |
| FYN |  |
| SHC2 |  |
| SHC4 |  |
| SHC3 |  |
| SHC1 |  |
| GRB2 |  |
| SOS1 |  |
| SOS2 |  |
| ARAF |  |
| BRAF |  |
| RAF1 |  |
| HCST |  |
| CD244 |  |
| PRKCG |  |
| SH2D1B |  |
| FAS |  |
| GZMB |  |
| PRF1 |  |
| CASP3 |  |
| CD3D |  |
| CD3E |  |
| CD3G |  |
| ITK |  |
| TEC |  |
| NCK1 |  |
| NCK2 |  |
| GRAP2 |  |
| PAK2 |  |
| PAK3 |  |
| PAK4 |  |
| PAK6 |  |
| PAK5 |  |
| RHOA |  |
| CD28 |  |
| ICOS |  |
| CBLC |  |
| CBLB |  |
| CDK4 |  |
| RASGRP1 |  |
| PDK1 |  |
| PRKCQ |  |
| TRAC |  |
| TRAJ1 |  |
| TRAJ2 |  |
| TRAJ3 |  |
| TRAJ4 |  |
| TRAJ5 |  |
| TRAJ6 |  |
| TRAJ7 |  |
| TRAJ8 |  |
| TRAJ9 |  |
| TRAJ10 |  |
| TRAJ11 |  |
| TRAJ12 |  |
| TRAJ13 |  |
| TRAJ14 |  |
| TRAJ15 |  |
| TRAJ16 |  |
| TRAJ17 |  |
| TRAJ18 |  |
| TRAJ19 |  |
| TRAJ20 |  |
| TRAJ21 |  |
| TRAJ22 |  |
| TRAJ23 |  |
| TRAJ24 |  |
| TRAJ25 |  |
| TRAJ26 |  |
| TRAJ27 |  |
| TRAJ28 |  |
| TRAJ29 |  |
| TRAJ30 |  |
| TRAJ31 |  |
| TRAJ32 |  |
| TRAJ33 |  |
| TRAJ34 |  |
| TRAJ35 |  |
| TRAJ36 |  |
| TRAJ37 |  |
| TRAJ38 |  |
| TRAJ39 |  |
| TRAJ40 |  |
| TRAJ41 |  |
| TRAJ42 |  |
| TRAJ43 |  |
| TRAJ44 |  |
| TRAJ45 |  |
| TRAJ46 |  |
| TRAJ47 |  |
| TRAJ48 |  |
| TRAJ49 |  |
| TRAJ50 |  |
| TRAJ52 |  |
| TRAJ53 |  |
| TRAJ54 |  |
| TRAJ56 |  |
| TRAJ57 |  |
| TRAJ58 |  |
| TRAJ59 |  |
| TRAJ61 |  |
| TRAV1-1 |  |
| TRAV1-2 |  |
| TRAV2 |  |
| TRAV3 |  |
| TRAV4 |  |
| TRAV5 |  |
| TRAV7 |  |
| TRAV8-1 |  |
| TRAV8-2 |  |
| TRAV8-3 |  |
| TRAV8-4 |  |
| TRAV8-6 |  |
| TRAV8-7 |  |
| TRAV9-1 |  |
| TRAV9-2 |  |
| TRAV10 |  |
| TRAV12-1 |  |
| TRAV12-2 |  |
| TRAV12-3 |  |
| TRAV13-1 |  |
| TRAV13-2 |  |
| TRAV14DV4 |  |
| TRAV16 |  |
| TRAV17 |  |
| TRAV18 |  |
| TRAV19 |  |
| TRAV20 |  |
| TRAV21 |  |
| TRAV22 |  |
| TRAV23DV6 |  |
| TRAV24 |  |
| TRAV25 |  |
| TRAV26-1 |  |
| TRAV26-2 |  |
| TRAV27 |  |
| TRAV29DV5 |  |
| TRAV30 |  |
| TRAV34 |  |
| TRAV35 |  |
| TRAV36DV7 |  |
| TRAV38-1 |  |
| TRAV38-2DV8 |  |
| TRAV39 |  |
| TRAV40 |  |
| TRAV41 |  |
| TRBC1 |  |
| TRBC2 |  |
| TRBD1 |  |
| TRBD2 |  |
| TRBJ1-1 |  |
| TRBJ1-2 |  |
| TRBJ1-3 |  |
| TRBJ1-4 |  |
| TRBJ1-5 |  |
| TRBJ1-6 |  |
| TRBJ2-1 |  |
| TRBJ2-2 |  |
| TRBJ2-3 |  |
| TRBJ2-4 |  |
| TRBJ2-5 |  |
| TRBJ2-6 |  |
| TRBJ2-7 |  |
| TRBV2 |  |
| TRBV3-1 |  |
| TRBV4-1 |  |
| TRBV4-2 |  |
| TRBV4-3 |  |
| TRBV5-1 |  |
| TRBV5-4 |  |
| TRBV5-5 |  |
| TRBV5-6 |  |
| TRBV5-7 |  |
| TRBV5-8 |  |
| TRBV6-1 |  |
| TRBV6-2 |  |
| TRBV6-3 |  |
| TRBV6-4 |  |
| TRBV6-5 |  |
| TRBV6-6 |  |
| TRBV6-7 |  |
| TRBV6-8 |  |
| TRBV6-9 |  |
| TRBV7-2 |  |
| TRBV7-3 |  |
| TRBV7-4 |  |
| TRBV7-6 |  |
| TRBV7-7 |  |
| TRBV7-8 |  |
| TRBV7-9 |  |
| TRBV9 |  |
| TRBV10-1 |  |
| TRBV10-2 |  |
| TRBV10-3 |  |
| TRBV11-1 |  |
| TRBV11-2 |  |
| TRBV11-3 |  |
| TRBV12-3 |  |
| TRBV12-4 |  |
| TRBV12-5 |  |
| TRBV13 |  |
| TRBV14 |  |
| TRBV15 |  |
| TRBV16 |  |
| TRBV17 |  |
| TRBV18 |  |
| TRBV19 |  |
| TRBV20-1 |  |
| TRBV24-1 |  |
| TRBV25-1 |  |
| TRBV27 |  |
| TRBV28 |  |
| TRBV29-1 |  |
| TRBV30 |  |
| TRDC |  |
| TRDD1 |  |
| TRDD2 |  |
| TRDD3 |  |
| TRDJ1 |  |
| TRDJ2 |  |
| TRDJ3 |  |
| TRDJ4 |  |
| TRDV1 |  |
| TRDV2 |  |
| TRDV3 |  |
| TRGV9 |  |
| TRGV8 |  |
| TRGV5 |  |
| TRGV4 |  |
| TRGV3 |  |
| TRGV2 |  |
| TRGJP2 |  |
| TRGJP1 |  |
| TRGJP |  |
| TRGJ2 |  |
| TRGJ1 |  |
| TRGC2 |  |
| TRGC1 |  |
| TRAV6 |  |

**Table S3. Gene Set Enrichment Analysis (GSEA) of different OMIRPS subgroups regarding Hallmark signaling pathways in the TCGA cohort.**

| ID | Description | setSize | enrichmentScore | NES | pvalue | p.adjust | qvalues |
| --- | --- | --- | --- | --- | --- | --- | --- |
| HALLMARK_E2F_TARGETS | HALLMARK_E2F_TARGETS | 200 | -0.590285168 | -2.4122822 | 1.00E-10 | 8.33E-10 | 4.56E-10 |
| HALLMARK_MYC_TARGETS_V2 | HALLMARK_MYC_TARGETS_V2 | 58 | -0.662327289 | -2.2502622 | 5.03E-06 | 1.48E-05 | 8.09E-06 |
| HALLMARK_MYC_TARGETS_V1 | HALLMARK_MYC_TARGETS_V1 | 199 | -0.539449128 | -2.2042933 | 2.66E-09 | 1.48E-08 | 8.09E-09 |
| HALLMARK_OXIDATIVE_PHOSPHORYLATION | HALLMARK_OXIDATIVE_PHOSPHORYLATION | 199 | -0.535105727 | -2.1865454 | 4.87E-09 | 2.43E-08 | 1.33E-08 |
| HALLMARK_KRAS_SIGNALING_DN | HALLMARK_KRAS_SIGNALING_DN | 136 | -0.511212677 | -1.9695677 | 4.13E-06 | 1.29E-05 | 7.07E-06 |
| HALLMARK_G2M_CHECKPOINT | HALLMARK_G2M_CHECKPOINT | 198 | -0.479009511 | -1.9585307 | 1.12E-06 | 4.00E-06 | 2.19E-06 |
| HALLMARK_FATTY_ACID_METABOLISM | HALLMARK_FATTY_ACID_METABOLISM | 147 | -0.345606551 | -1.3488844 | 0.02254267 | 0.04900581 | 0.02682423 |
| HALLMARK_ADIPOGENESIS | HALLMARK_ADIPOGENESIS | 194 | 0.377901417 | 1.34809722 | 0.03583276 | 0.07465159 | 0.04086192 |
| HALLMARK_IL2_STAT5_SIGNALING | HALLMARK_IL2_STAT5_SIGNALING | 190 | 0.391300278 | 1.39454559 | 0.01734169 | 0.03941292 | 0.02157339 |
| HALLMARK_APOPTOSIS | HALLMARK_APOPTOSIS | 157 | 0.429888497 | 1.49941415 | 0.00898868 | 0.02247171 | 0.0123003 |
| HALLMARK_HEDGEHOG_SIGNALING | HALLMARK_HEDGEHOG_SIGNALING | 34 | 0.584720346 | 1.58753603 | 0.01043016 | 0.02483372 | 0.01359319 |
| HALLMARK_COMPLEMENT | HALLMARK_COMPLEMENT | 186 | 0.461155718 | 1.64038683 | 0.00046762 | 0.00129896 | 0.00071101 |
| HALLMARK_TGF_BETA_SIGNALING | HALLMARK_TGF_BETA_SIGNALING | 52 | 0.588365486 | 1.75615208 | 0.00194036 | 0.00510621 | 0.00279498 |
| HALLMARK_HYPOXIA | HALLMARK_HYPOXIA | 188 | 0.541891772 | 1.93223467 | 1.32E-07 | 5.49E-07 | 3.01E-07 |
| HALLMARK_INFLAMMATORY_RESPONSE | HALLMARK_INFLAMMATORY_RESPONSE | 186 | 0.544378116 | 1.93641899 | 2.67E-07 | 1.03E-06 | 5.61E-07 |
| HALLMARK_TNFA_SIGNALING_VIA_NFKB | HALLMARK_TNFA_SIGNALING_VIA_NFKB | 198 | 0.577101815 | 2.06550293 | 2.61E-09 | 1.48E-08 | 8.09E-09 |
| HALLMARK_ANGIOGENESIS | HALLMARK_ANGIOGENESIS | 34 | 0.761940501 | 2.0686949 | 1.69E-06 | 5.64E-06 | 3.09E-06 |
| HALLMARK_KRAS_SIGNALING_UP | HALLMARK_KRAS_SIGNALING_UP | 187 | 0.594924626 | 2.11909965 | 2.23E-10 | 1.59E-09 | 8.71E-10 |
| HALLMARK_PANCREAS_BETA_CELLS | HALLMARK_PANCREAS_BETA_CELLS | 29 | 0.830157719 | 2.18776149 | 5.27E-08 | 2.40E-07 | 1.31E-07 |
| HALLMARK_APICAL_JUNCTION | HALLMARK_APICAL_JUNCTION | 183 | 0.621411747 | 2.20426149 | 1.00E-10 | 8.33E-10 | 4.56E-10 |
| HALLMARK_COAGULATION | HALLMARK_COAGULATION | 116 | 0.686256686 | 2.2977611 | 1.00E-10 | 8.33E-10 | 4.56E-10 |
| HALLMARK_UV_RESPONSE_DN | HALLMARK_UV_RESPONSE_DN | 140 | 0.671140134 | 2.30903208 | 1.00E-10 | 8.33E-10 | 4.56E-10 |
| HALLMARK_MYOGENESIS | HALLMARK_MYOGENESIS | 157 | 0.727461582 | 2.5373235 | 1.00E-10 | 8.33E-10 | 4.56E-10 |
| HALLMARK_EPITHELIAL_MESENCHYMAL_TRANSITION | HALLMARK_EPITHELIAL_MESENCHYMAL_TRANSITION | 198 | 0.808747727 | 2.89458595 | 1.00E-10 | 8.33E-10 | 4.56E-10 |
